# Identification and analysis of the crucial holin domain and sites and the bactericidal activity of a holin–endolysin lysis cassette from phage PZL-Ah152 against *Aeromonas hydrophila*

**DOI:** 10.1128/jvi.00832-25

**Published:** 2025-12-15

**Authors:** Chao Feng, Yan Cheng, Shuang Liang, Ruiqi Liang, Jiahao Yu, Shun Wang, Hui Guo, Xiaofeng Shan, Dongxing Zhang, Aidong Qian, Wuwen Sun, Lei Zhang

**Affiliations:** 1 College of Animal Science and Technology, Jilin Agricultural University 85112, Changchun, China; 2 Ninth People's Hospital Affiliated to Shanghai Jiaotong University School of Medicine 56694, Shanghai, China; 3 Borui Technology Co., Ltd., Changchun, China; Michigan State University, East Lansing, Michigan, USA

**Keywords:** phage, holin, lysin, fusion protein, mechanism of action

## Abstract

With the increasing prevalence of antibiotic-resistant bacteria, phage therapy has garnered significant attention. Holin and lysin play essential roles in the phage-induced lysis of bacteria. This study investigated the functions of the holin protein Hol 46 and the lysozyme protein Lys 17 from the phage PZL-Ah152 and the mechanisms underlying the action of the fusion protein Hol 46_Lys 17. Assays, including membrane protein extraction tests and fluorescence microscopy, verified that the Hol 46 protein localized to the cell membrane and significantly inhibited the growth of *Aeromonas hydrophila* Ah152. We determined that the Hol 46 C-terminal domain and glutamic acid residue at position 66, along with the lysine residues at positions 63 and 64, were critical for its cell-penetrating activity. Furthermore, the Hol 46_Lys 17 fusion protein was developed to combine the membrane-disrupting capacity of Hol 46 with the lytic action of Lys 17. Hol 46_Lys 17 exhibited not only broader antibacterial effects against *Aeromonas* (24/38) but also against *Escherichia coli* (3/12) and *Salmonella* (5/29). Transcriptomic studies revealed that treatment with Hol 46_Lys 17 led to significant changes in the expression of genes related to flagellar synthesis, bacterial chemotaxis, and TCSs. Finally, animal studies were performed to confirm the safety and effectiveness of Hol 46_Lys 17 in treating intestinal infections caused by *A. hydrophila* in crucian carp. Our data showed that phage lytic system-related proteins hold great potential for the treatment of infections caused by antibiotic-resistant bacteria.

**IMPORTANCE:**

As a zoonotic and fish-pathogenic bacterium, *Aeromonas hydrophila* causes significant harm worldwide. Owing to the emergence of *A. hydrophila* strains with multidrug resistance, phage therapy has garnered extensive attention. Holin and lysin, phage-derived antibacterial proteins, play crucial roles in antimicrobial activity. The glutamic acid at position 66 and lysine residues at positions 63 and 64 in the C-terminal domain of the Hol 46 protein from phage PZL-Ah152 were essential for its *A. hydrophila* cell-penetrating activity. The Hol 46_Lys 17 fusion protein exhibited broad-spectrum antibacterial activity, including effects against *Salmonella* and *Escherichia coli*. Transcriptomic assays further revealed the effects of Hol 46_Lys 17 on *A. hydrophila* Ah152 at the molecular level. *In vivo* studies confirmed its efficacy and safety for the treatment of intestinal infections in crucian carp.

## INTRODUCTION

With the growing severity of bacterial resistance to antibiotics, environmental concerns resulting from antibiotic misuse have drawn considerable attention in recent years. The identification of alternatives to traditional antibiotics has become a major public health challenge. As a prevalent aquatic pathogen, *Aeromonas hydrophila (A. hydrophila*) presents severe challenges to freshwater aquaculture practices worldwide (1). Animals infected with *A. hydrophila* typically exhibit symptoms such as skin ulcers and bleeding, and severe infections can lead to septicemia or even death (2). Notably, this opportunistic pathogen exhibits zoonotic potential through its tripartite transmission cycle, causing acute gastroenteritis in immunocompromised humans while maintaining environmental reservoirs in aquatic ecosystems (3, 4). Currently, antibiotic therapy remains the primary strategy for controlling and eradicating *A. hydrophila* infections. However, the emergence of antibiotic-resistant strains has led to a notable decline in treatment efficacy (5). In recent years, phage therapy has garnered attention as a novel antibacterial treatment method (6, 7).

As viruses that specifically target bacteria, bacteriophages are increasingly favored for their high specificity and potency against host strains. However, phage therapy also faces many challenges, such as high specificity, low stability, the standardization of clinical applications, and the development of resistance (8). Recently, phage-derived protein therapy has attracted significant attention as a novel antibacterial treatment method (9). Phage proteins, which are released following phage infection, exhibit potent antibacterial activity (10). Research on phage proteins in mammalian systems is relatively extensive, and some studies have also been conducted in fish. Bacteriophage proteins can specifically disrupt bacterial cell walls or cell membranes, thereby inhibiting bacterial growth and spread (11, 12). Compared with conventional antibiotics, phage protein therapy presents distinct advantages. First, the highly specific nature of phage proteins allows for precise targeting of bacteria that cause infection, thereby minimizing harm to the host’s commensal microbiota (13). Moreover, compared with broad-spectrum antibiotics, phage-derived antibacterial agents have greater species or strain specificity, which minimizes their potential impact on the gut microbiota (14). Second, as phage proteins originate from natural phages, they are less likely to induce the development of resistance (15). Additionally, phage proteins exhibit lower toxicity and fewer side effects, which can help decrease adverse reactions during treatment (16). Previous studies have also reported that even upon repeated exposure, lysozyme does not cause significant adverse effects on the host immune response (17). Recent studies have demonstrated the potential of phage protein therapy in antibacterial applications, showing not only effective antibacterial activity *in vitro* but also promising results in animal models and preliminary clinical trials (18).

Holin is a protein derived from phages that disrupts bacterial cell membranes (19). It specifically targets the membranes of certain bacteria, resulting in their rupture and lysis, ultimately compromising bacterial integrity and leading to cell death (20). To date, more than 250 types of phage holin proteins have been identified; however, while these proteins share similar functions, their homology is relatively low, and they display considerable structural diversity. On the basis of the topology of their transmembrane α-helical segments, phage holin proteins can be categorized into three classes (21). Class I holins are characterized by three transmembrane α-helices arranged in an N-terminal-to-C-terminal topology, whereas class II holins feature two transmembrane α-helices with a similar topological organization. Examples include the class I holin S105 of lambda and the class II holin S^21^68 of lambdoid bacteriophage ϕ21 (22). In contrast, class III holins typically exhibit a single transmembrane α-helix with either an N-terminal or C-terminal topological orientation and serve as critical components of T4-like and T5-like phages (21). Phage lysin facilitates the penetration and dissolution of bacterial cell walls, enabling the release of the phage’s genome and allowing replication inside the bacterial cell (23). Given their unique properties, phage lysins have been actively studied and applied across various fields, including biotechnology and medicine, as antibacterial agents against resistant strains (24).

In this work, we investigated the holin protein Hol 46 from the *Aeromonas hydrophila* phage PZL-Ah152, which has shown excellent antibacterial effects against antibiotic-resistant bacteria, with a focus on its mechanism of gram-negative bacteria membrane perforation. Furthermore, a fusion protein, Hol 46_Lys 17, was successfully constructed. Comprehensive investigations of the biological characteristics and bactericidal mechanism and a transcriptomic analysis of Hol 46_Lys 17 were performed. Finally, animal experiments were conducted to validate the safety and efficacy of Hol 46_Lys 17 in treating intestinal infections caused by *A. hydrophila* in crucian carp. The application of phage-derived proteins provides an environmentally friendly solution to the problem of antibiotic-resistant bacterial infections.

## MATERIALS AND METHODS

### Bacterial strains and bacteriophages

*Aeromonas hydrophila* Ah152 was isolated from diseased fish and exhibited multidrug resistance (25). The phage PZL-Ah152 used in this study was isolated from sewage systems in Changchun, China, and stored in the Preventive Veterinary Laboratory of Jilin Agricultural University (25). The bacterium was preserved in 20% glycerin at −80°C. For experimental purposes, the original frozen bacteria were streaked on the LB solid medium and cultured at 37°C for 12 h. A single colony was then inoculated in liquid LB medium and cultured at 37°C (120 rpm) for 12 h to obtain purified *A. hydrophila* Ah152. Phage isolation and purification were performed using the double agar overlay method. Briefly, serially diluted phage lysates were mixed with mid-log phase *A. hydrophila* Ah152 (optical density of 0.6 [OD_600_]) in 0.7% soft agar and overlaid onto 1.5% LB agar plates. After 18 h of incubation at 37°C, a single well-isolated plaque was excised and eluted in phosphate-buffered saline (PBS). The eluate was filtered (0.22 µm) and propagated by infecting fresh *A. hydrophila* cultures at a multiplicity of infection (MOI) of 0.1. Following lysis (37°C, 6 h), the debris was removed by centrifugation (8,000 × *g*, 10 min, 4°C). Three iterative rounds of plaque purification were conducted to ensure clonal phage homogeneity. In this study, 13 strains of *Aeromonas* that had been maintained in the laboratory of Jilin Agricultural University (2 strains of *A. caviae* isolated from diseased silver carp, 5 strains of *A. veronii* isolated from diseased carp, and 6 strains of *A. hydrophila* isolated from yellow catfish) were used, in addition to 66 strains that were donated by the Changchun Veterinary Research Institute, Chinese Academy of Agricultural Sciences, Changchun, China (3 strains of *A. caviae*, 22 strains of *A. hydrophila*, 12 strains of *Escherichia coli*, and 29 strains of *Salmonella*).

### Bioinformatics analysis

The ClustalX 2.0.12 program was used to compare highly homologous protein sequences. The conservation of the Lys 17 protein was analyzed with the WebLog online tool (https://weblogo.berkeley.edu/). The TMHMM server v. 2.0 (http://www.cbs.dtu.dk/services/TMHMM/), PROTTER (https://wlab.ethz.ch/protter/start/), and TOPCONS (https://topcons.cbr.su.se) were used to predict the transmembrane domain (TMD), coiled coils, signal peptides, and other biological features of the Hol 46 protein. Additionally, the protein sequence was submitted to the Predicting Antigenic Peptides tool (http://imed.med.ucm.es/Tools/antigenic.pl) for secondary structure prediction.

### *In vitro* tests

#### Protein construction and expression

The genomic sequence of phage PZL-Ah152 (GenBank: MW671054.1), previously characterized in our laboratory, was predicted to encode Holin (ORF46, QTH79787.1) and Lysin (ORF17, QTH79758.1) proteins (25). To investigate their functional roles, ORF46 and ORF17 fragments were amplified by PCR using the phage genome as a template. Full-length (Hol 46), truncated, and mutated variants of ORF46 were generated by PCR (the primers and conditions used are listed in Table S1), and site-directed mutagenesis was performed using the Mut Express II Fast Mutagenesis Kit V2 (Vazyme Biotech Co., Ltd.). The N-terminal His tag of the double-tagged Hol 46 (Hol 46 NC) was derived from the intrinsic 6 × His tag of the pET-28a vector, which was located upstream of the multiple cloning site (MCS) and was fused in-frame with the target gene upon insertion. For the C-terminal His tag, we designed specific forward and reverse primers to introduce a 6 × His coding sequence (5′- GTGGTGGTGGTGGTGGTG −3′) immediately downstream of the ORF46-coding region while deleting the native stop codon of ORF46 via PCR amplification. The PCR-amplified ORF46 fragment (with the stop codon deleted and C-terminal 6 × His sequence added) was cloned and inserted into the pET-28a vector, resulting in the recombinant plasmid pET-28a-Hol 46 NC. The fusion protein constructs were engineered via overlap PCR using the templates ORF46 and ORF17 (Table S1). For subcellular localization analysis, GFP was fused in-frame to either Hol 46 or Hol (39–67) through primer-mediated linkage (Table S1), enabling fluorescence microscopy analysis. The λ holin S105 protein was constructed according to a previous method (26).

All the constructs were directionally cloned and inserted into the pET-28a vector MCS using the ClonExpress II One-Step Cloning Kit (Vazyme Biotech Co., Ltd.). The recombinant plasmids were transformed into *E. coli* BL21(DE3)-competent cells via chemical transformation. Positive clones were selected on LB agar supplemented with 50 µg/mL kanamycin, verified by colony PCR and sequencing.

For protein expression, cultures were induced with IPTG (final concentration: 1 mM) at an OD_600_ ≈ 0.5 and incubated for 4 h at 37°C. Cells were harvested by centrifugation (8,000 × *g*, 10 min, 4°C), resuspended in PBS (for membrane proteins, the bacterial cell pellet was incubated with ice-cold 0.1% Triton X-100 in PBS for 30 min at 4°C), and lysed via sonication (3 × 30 s pulses at 40% amplitude). The supernatant (the soluble fraction of the whole-cell lysates) was loaded onto a pre-equilibrated gravity column containing Ni Sepharose 6 Fast Flow resin (Cytiva). After washing the column with 10 column volumes of wash buffer (20 mM Tris-HCl, 500 mM NaCl, and 10 mM imidazole; pH 7.9), the 6 × His-tagged proteins were eluted with elution buffer (20 mM Tris-HCl, 500 mM NaCl, and 300 mM imidazole; pH 7.9). The eluate was concentrated using Amicon Ultra centrifugal filters (10 kDa MWCO; Merck Millipore) and subjected to buffer exchange to remove imidazole (the membrane proteins were dialyzed against PBS). Protein purity was verified by 12% SDS‒PAGE. For immunoblotting analysis, proteins were transferred to PVDF membranes at 200 mA for 30 min. The membranes were probed with an anti-His tag monoclonal antibody (1:5,000; Solarbio) followed by a HRP-conjugated goat anti-mouse IgG (1:10,000; Solarbio). The signals were detected using an ECL luminol substrate.

#### Protein antimicrobial activity

The concentration of *A. hydrophila* Ah152 was adjusted to 1 × 10^8^ CFU/mL. The concentrations of Hol 46 and Hol 46_Lys 17 were 0.6 µg/µL, and the concentrations of Lys 17 were 0.6 µg/µL, 0.3 µg/µL, 0.15 µg/µL, and 0.075 µg/µL. Each protein solution was mixed with the *A. hydrophila* Ah152 suspension at a 1:1 (vol/vol) ratio in 10 mL sterile test tubes (the same volume of PBS was used in the control group). Each mixture was incubated at 37°C for 9 h, and the cells were counted at 1, 2, 3, 6, and 9 h. Each group contained three replicates.

The antimicrobial activity of Hol 46 and its truncated/mutated variants was evaluated using a modified agar well diffusion assay (27). *A. hydrophila* Ah152 cultures were standardized to a concentration of 1 × 10^8^ CFU/mL in sterile PBS. A 200 µL aliquot of the bacterial suspension was uniformly mixed with 10 mL of molten semisolid LB agar (0.75% wt/vol agar) and overlaid onto LB solid agar plates pre-embedded with sterile Oxford cups. After solidification at room temperature, the Oxford cups were carefully removed, leaving uniform cylindrical wells. Each well was loaded with 100 µL of the following test samples (all adjusted to 0.6 µg/µL in PBS): Hol 46 full-length protein, truncated variants (Hol [1–57], Hol [39–67], and Hol [ΔTMD]), and point mutants (Hol [61D], Hol [58D], Hol [63D], Hol [64D], Hol [65R], Hol [66R], Hol [61,63,64D], Hol [65,66R], and Hol [63,64D]); the supernatant of the pET-28a-BL21 (empty vector expression) was used in the control group. The plates were incubated at 37°C for 12 h, after which the diameters of the inhibition zones were measured using digital calipers. All experiments were performed with three biological replicates.

A UV‒visible spectrophotometer was used to assess the effects of Hol 46 and Hol (39–67) on *A. hydrophila* Ah152. An *A. hydrophila* Ah152 bacterial suspension (100 µL) was inoculated into an LB liquid culture to achieve an OD_600_ of 0.5. Afterward, Hol 46 (0.6 µg/μL; 100 µL) and Hol (39–67) (0.6 µg/µL, 100 µL) were added to the *A. hydrophila* Ah152 suspension, which was subsequently added to a 96-well plate. PBS was used as a control. The mixture was incubated in a 37°C incubator for 120 min, and the A_260_ values were measured at 10, 20, 40, 60, 80, 100, and 120 min. Each sample consisted of three replicates.

#### Scanning electron microscopy

*A. hydrophila* Ah152 was cultured to the logarithmic growth phase, and the concentration was adjusted to 1 × 10^8^ CFU/mL. The concentrations of the Lys 17 and Hol 46_Lys 17 protein supernatants were adjusted to 0.6 µg/µL. A mixture of 100 µL of the bacterial suspension and 100 µL of Hol 46 protein was incubated in a 37°C incubator for 3 h, and PBS was used as the control. The bacteria were subsequently fixed with glutaraldehyde and dehydrated in a graded ethanol series for 20 min. The samples were dried overnight at room temperature. The dried samples were prepared for SEM analysis (Field Emission Scanning Electron Microscope SEM 4000 Pro, JEOL Ltd., Japan).

#### β-Galactosidase activity

ONPG, a substrate of β-galactosidase, can be used to measure the activity of β-galactosidase in bacterial suspensions and assess cell membrane permeability. Bacteria were cultured to the logarithmic phase and centrifuged at 2,000 × *g* for 10 min, after which the supernatant was discarded. The pellet was subsequently washed three times with 0.05 mol/L NaH_2_PO_4_ buffer and then resuspended. The resuspended bacterial cells were treated with Hol 46 or Hol (39–67) (0.6 µg/µL) (PBS was used as a control). Afterward, 5 mL of the suspension was removed and centrifuged at 5,500 × *g* for 15 min. After centrifugation, 1 mL of the supernatant was collected and mixed with 4 mL of 0.05 mol/L ONPG, followed by incubation in a 37°C water bath for 60 min. Afterward, 5 mL of 0.5 mol/L Na_2_CO_3_ was added to terminate the reaction. A 200 µL aliquot of each sample was transferred to a 96-well plate, and the absorbance at 420 nm (A_420_) was measured. The experiment was repeated three times.

#### Membrane potential detection

To evaluate the effects of Hol 46 and Hol (39–67) on the bacterial membrane potential, a modified Rhodamine 123 (Rh123) fluorescence assay was performed as previously described (28). *A. hydrophila* Ah152 was cultured to the mid-log phase (OD_600_ = 0.6) at 37°C with shaking, harvested by centrifugation (2,000 × *g*, 10 min), and washed three times with 10 mM PBS (pH 7.4). Bacterial cells were resuspended in PBS and treated with Hol 46, Hol 46 NC, or Hol (39–67) (0.6 µg/µL) for 1 h at 37°C (controls received equivalent volumes of PBS). The uncoupler carbonyl cyanide m‐chlorophenyl hydrazone (10 µmol/L) was used to disrupt the cells during the experiment. λ phage holin S105 (a verified holin) was used as a control (26). Rh123 (1 mg/mL stock solution in PBS) was added to the cell suspensions to a final concentration of 2 µg/mL. After 30 min of incubation in the dark, the cells were washed twice with PBS, resuspended, and transferred to a 10 mm quartz cuvette. The fluorescence intensity was measured using a Hitachi F-4500 spectrofluorometer (Hitachi, Japan) at excitation/emission wavelengths of 480/530 nm. Data from triplicate experiments were expressed as the mean fluorescence intensity. To assess the activity in the expression host BL21, *E. coli* strains pET-28a-BL21 (empty vector) or pET-28a-Hol 46-BL21, pET-28a-S105-BL21 and pET-28a-Hol 46 NC-BL21 were grown to OD_600_ = 0.6, induced with IPTG (final concentration: 1 mM) for 4 h (uninduced Hol 46-BL21 served as a control), washed, resuspended, and subjected to Rh123 fluorescence measurements as described above.

#### Flow cytometry

*A. hydrophila* Ah152 was cultured to the logarithmic growth phase, centrifuged, washed, and resuspended in PBS. Afterward, Hol 46 or Hol (39–67) was added to a final concentration of 0.6 µg/µL and incubated at 37°C, and PBS was used as a control. The pET-28a-Hol 46-BL21 strain was cultured to an OD_600_ of 0.5, IPTG was subsequently added to a final concentration of 1 mM, and the culture was incubated for 4 h to induce protein expression. A control group to which IPTG was not added was also included in the experiment. Afterward, propidium iodide (PI) staining solution was added to the culture at a final concentration of 25 µg/mL, and the mixture was incubated in the dark at 37°C for 10 min, followed by centrifugation. The excess PI was removed by washing with PBS, and the cells were resuspended in 200 µL of PBS. The number of bacterial cells with positive PI staining was detected via flow cytometry (FACS). Each sample consisted of three replicates. When investigating the function of Hol (39–67), the Ah152 group was used as a negative control, and the Ah152 +Hol 46 group was used as a positive control for analysis.

#### Subcellular localization

The strains GFP-pET-28a-Hol 46-BL21, GFP-pET-28a-Hol (39–67)-BL21, and the control GFP-pET-28a-BL21 were each cultured at a 1% ratio in LB medium and incubated for 5 h. An IPTG solution was added to a final concentration of 1 mmol/L (with a no IPTG group as a control) and incubated for 4 h to induce protein expression. From each group, 1 mL of culture was centrifuged at 2,000 × *g* for 10 minutes. The pellet was then resuspended in 300 µL of PBS. Next, 10 µL of the induced bacterial culture was added to 100 µL of FM4-64 solution, and the mixture was incubated on ice for 1 min and observed with a fluorescence microscope (each sample consisted of three replicates).

The strains pET-28a-Hol 46-BL21, GFP-pET-28a-Hol (39–67)-BL21, and the control pET-28a-BL21 were each subcultured at a 1% ratio in LB liquid medium and induced with IPTG (1 mM), and after 5 h of induction, the cells were collected via centrifugation (3,000 × *g*, 10 min, 4°C). Total proteins, cytosolic proteins, and membrane proteins were extracted using a Membrane and Cytosol Protein Extraction Kit (Beyotime Biotechnology, Cat# P0033) according to the manufacturer’s protocol. Briefly, cells were lysed in ice-cold lysis buffer containing protease inhibitors (1 mM PMSF) and centrifuged at 12,000 × *g* for 15 min at 4°C to separate the cytosolic (supernatant) and membrane (pellet) fractions. Protein concentrations were determined via a BCA assay. Equal amounts of protein (20 µg per lane) were resolved by 12% SDS‒PAGE and transferred to PVDF membranes.

#### Effect of EDTA on Lys 17

*A. hydrophila* Ah152 in the logarithmic growth phase was adjusted to 5 × 10^8^ CFU/mL. An initial EDTA solution (10 mM) was used to prepare *A. hydrophila* Ah152 suspensions (1 × 10^8^ CFU/mL) with final EDTA concentrations of 0.5 mM, 1 mM, 1.5 mM, and 2 mM. Similarly, a 100 mM citric acid solution was used to prepare *A. hydrophila* Ah152 suspensions (1 × 10^8^ CFU/mL) with final citric acid concentrations of 1 mM, 2 mM, 5 mM, and 10 mM, which were incubated in a 37°C incubator for 30 min. Next, Lys 17 (50 μg) was added to each of the above samples, which were incubated for an additional hour before the colonies were counted. To verify the effect of EDTA on *A. hydrophila* Ah152, a control group without the Lys 17 protein was prepared, and *A. hydrophila* Ah152 incubated with PBS instead of EDTA served as the positive control. Each sample consisted of three replicates.

#### Activity of Hol 46_Lys 17

The lytic activity of Hol 46_Lys 17 was quantitatively assessed using a modified turbidimetric assay (29). Briefly, *A. hydrophila* Ah152 suspensions were adjusted to an optical density of 0.6 (OD_600_) in fresh LB medium. The Hol 46_Lys 17 protein was serially diluted to concentrations of 0.6, 0.3, 0.15, 0.075, and 0.0375 µg/µL in 10 mM PBS. For the assay, 100 µL of each protein dilution was mixed with 100 µL of the bacterial suspension in a 96-well microplate. The plate was incubated at 37°C for 30 min in a spectrophotometric microplate reader, with OD_600_ measurements recorded at 1 min intervals. Relative activity was defined as the ratio of the decrease in the bacterial turbidity over 30 min to the average decrease in the turbidity. Each sample consisted of three replicates.

#### Evaluation of the bactericidal activity of the phage proteins

The host ranges of Hol 46_Lys 17 (0.6 μg/μL), Hol 46 (0.6 μg/μL), Lys 17 (0.6 μg/μL), Hol 46 + Lys 17 (0.6 μg/μL) and the phage PZL-Ah152 (1 × 10^7^ PFU/mL) were confirmed via a spot assay, performed similarly to previous methods (25). These assays included a total of 28 strains (1 × 10^8^ CFU/mL) of *A. hydrophila*, 5 strains of *A. veronii*, 5 strains of *A. caviae*, 12 strains of *E*. *coli*, and 29 strains of *Salmonella*. Each sample consisted of three replicates.

#### Biofilm assay

The 96-well microtiter plate method was used in this study with minor modifications (30). In brief, we stained biofilms with crystal violet in a 96-well microplate to observe the ability of the phage to remove the biofilm *in vitro*. Bacteria in the logarithmic growth stage were diluted (20 µL of bacterial suspension and 180 µL of LB medium) in 96-well plates and incubated at 37°C for 48 h. After the biofilms were washed three times, Hol 46 (0.6 μg/μL, 100 µL), Lys 17 (0.6 μg/μL, 100 µL), Hol 46 (0.6 μg/μL, 50 µL)+Lys 17 (0.6 μg/μL, 50 µL), or Hol 46_Lys 17 (0.6 μg/μL, 100 µL) was added to the 96-well plate. The control group was established and cultured at 37°C for 6 h. The biofilms were stained with crystal violet. The OD_590nm_ was measured following glacial acetic acid decolorization using a full-wavelength microplate reader. Each sample consisted of three replicates.

#### Transcriptomic analysis

To determine an appropriate concentration of Hol 46_Lys 17 that permitted bacterial interaction without substantial bacteriostasis for transcriptomic analysis, a bacterial viability assay was conducted. *A. hydrophila* Ah152 cultures were standardized to 1 × 10^8^ CFU/mL. Aliquots (1 mL) of the bacterial suspension were incubated with 5 µg, 10 µg, 15 µg, 20 µg, or 25 µg of the Hol 46_Lys 17 protein (dissolved in PBS) at 37°C for 1 h. The negative control group received an equal volume of PBS. Serial dilutions were subsequently plated for colony counting to quantify viable bacteria. The Hol 46_Lys 17 concentration at which the cell viability exceeded 75% was selected as the optimal treatment concentration for the transcriptomic experiments. All treatments were performed with three biological replicates.

Total RNA was extracted from the bacterial samples using the RNeasy Protect Bacteria Midi Kit (Qiagen) following the manufacturer’s protocol. RNA integrity was evaluated via an Agilent 2100 Bioanalyzer (Agilent Technologies), and the nucleic acid concentration was quantified using a NanoDrop 2000 spectrophotometer (Thermo Scientific). RNA-seq library construction and high-throughput sequencing were conducted by Novogene (Beijing) using the Illumina TruSeq RNA Sample Prep Kit. Briefly, cDNA libraries were generated from qualified RNA samples, normalized, and sequenced on the Illumina NextSeq 500 platform (High Output Kit, Illumina) with three biological replicates per group.

For bioinformatics analysis, sequencing reads were aligned to the reference genome using Bowtie2 (v2.3.4.3). Gene expression quantification was performed with FeatureCounts (v1.5.0-p3) and HTseq (0.9.1), followed by variant detection using GATK4 (v4.1.0.0) and SNPEff (v4.3q). Differential gene expression analysis was conducted with DESeq2 (1.20.0), and Gene Ontology and Kyoto Encyclopedia of Genes and Genomes (KEGG) pathway functional enrichment analyses were performed using clusterProfiler (v3.8.1).

### *In vivo* experiment

For the safety evaluation, 100 µL of Hol 46_Lys 17 (0.15 μg/μL) was administered via intraperitoneal (IP) injection to each wild-type crucian carp for 7 consecutive days. The control group received an equal volume of sterile PBS via the same route of administration. Tissue sampling was performed on Day 0 (pretreatment) and Day 7 (post-treatment), with three fish sampled at each time point. Liver, spleen, kidney, and intestinal tissues were surgically excised for histopathological analysis via hematoxylin and eosin (H&E) staining and quantitative real-time PCR (qRT‒PCR) analysis. The mRNA expression levels of *TGF-β*, *IFN-γ*, *TNF-α*, *IL-1β,* and *IL-10* were quantified via qPCR. Each sample consisted of three replicates. The primers used for the immunity-related genes and *β-actin*, used as a housekeeping gene, are listed in Table S2.

To determine the minimal lethal dose (MLD), 10 crucian carp in each experimental group were injected intraperitoneally (i.p.) with *A. hydrophila* 152 (at concentrations of 1 × 10^5^, 1 × 10^6^, 1 × 10^7^, 1 × 10^8^, and 1 × 10^9^ CFU/mL; 100 µL/fish). The control group was not inoculated with bacteria (10 mM PBS, 100 µL/fish), and 2× MLD was used as the infective inoculum.

Crucian carp were infected with 2× MLD (2 × 10^9^ CFU/mL, 100 µL/fish) of *A. hydrophila* 152 (*n* = 10). One hour later, the carp were treated with 100 µL of Hol 46_Lys 17 (0.15 μg/μL), Hol 46 (0.6 μg/μL), Lys 17 (0.6 μg/μL), Hol 46 + Lys 17 (0.6 μg/μL), or PZL-Ah152 (2 × 10^10^ PFU/mL). The mortality rates of the fish were recorded every 12 h for 7 days.

The bacterial loads in the crucian carp gut were subsequently determined. Intestinal samples were collected from each experimental group at 12, 18, 24, and 30 h after treatment, weighed, and suspended in filter-sterilized PBS. The collected intestinal structure slurry was subsequently diluted. Afterward, 100 µL of each dilution was used to determine the bacterial loads. Each sample consisted of three replicates.

Histopathological analysis of the carp intestines from each group after 30 h of treatment was performed. Briefly, the crucian carp tissues were removed, placed in 4% formalin, stained with H&E, and analyzed via microscopy.

The mRNA expression levels of *TGF-β*, *IL-10*, *Occludin*, *Claudin4*, *Muc2,* and *Zo-1* in the guts of different groups were quantified via qPCR (each sample consisted of three replicates). The primers used for the immunity-related genes and *β-actin*, which was used as a housekeeping gene, are listed in Table S2.

### Statistical analysis

The statistical data obtained in this study were analyzed via one-way analysis of variance (ANOVA) or Student’s *t* tests. All the images were generated with GraphPad Prism 8.0 (GraphPad Software, USA). The error bars represent the standard deviation of the mean. ∗∗∗*P* < 0.001, and ∗∗∗∗*P* < 0.0001 indicate very significant differences. R software (version 4.4.1) was used for data analysis.

## RESULTS

### Hol 46 induced membrane damage, leading to bacterial cell death

**Fig 1:**
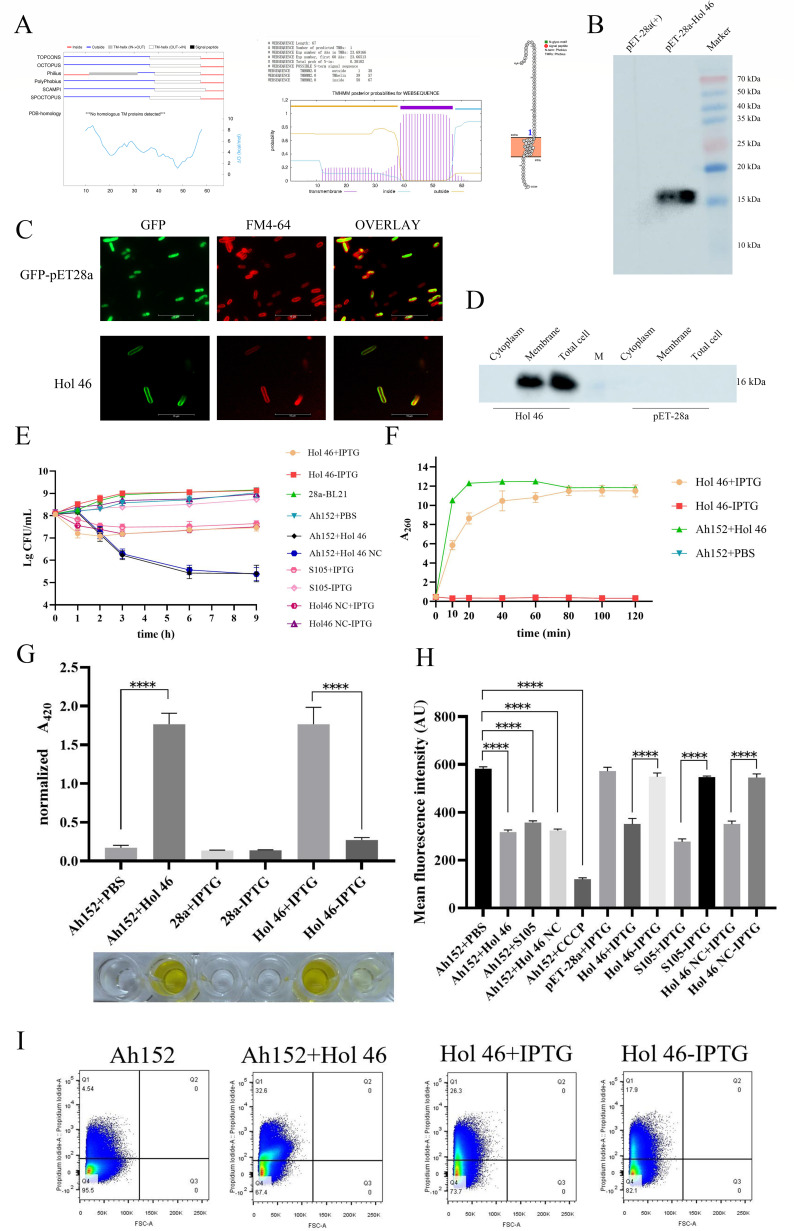
Hol 46 perforated the cell surface to promote bacterial cell lysis and death. (**A**) TOPCONS, TMHMM, and PROTTER predictions of the TMDs in Hol 46. (**B**) The expression of the Hol 46 protein in *E. coli* BL21(DE3) was analyzed by Western blotting. (**C**) Fluorescence microscopy images of *E. coli* BL21(DE3) expressing GFP-Hol 46 and GFP. (**D**) Subcellular localization of the protein Hol 46 in the expression strain *E. coli* BL21(DE3) determined by Western blotting. (**E**) Effects of Hol 46 on the survival rates of *E. coli* BL21(DE3) and *A. hydrophila* Ah152. (**F**) Quantification of extracellular nucleic acids was performed by UV spectroscopy. Supernatants from (i) IPTG-induced *E. coli* BL21(DE3) expressing Hol 46 and (ii) *A. hydrophila* Ah152 treated with the Hol 46 protein were analyzed for free nucleic acid content by measuring the absorbance at 260 nm. (**G**) β-Galactosidase activity assay. Enzymatic activity was quantified in (i) IPTG-induced *E. coli* BL21(DE3) expressing the Hol 46 protein and (ii) *A. hydrophila* Ah152 cultures treated with the Hol 46 protein. Cells were subjected to the ONPG hydrolysis assay. The activity was determined by measuring the A_420_.∗*P* < 0.05, ∗∗*P* < 0.01, ∗∗∗*P* < 0.001, ∗∗∗∗*P* < 0.0001. (**H**) Effect of the Hol 46 protein on the transmembrane potential of *A. hydrophila* Ah152 and *E. coli* BL21(DE3). (**I**) The integrity of the bacterial cell membrane was detected by FACS. The fluorescence intensity of PI was measured in (i) *E. coli* BL21(DE3) cells induced with IPTG to express the Hol 46 protein and (ii) *A. hydrophila* Ah152 cells treated with the Hol 46 protein.

The structure of the Hol 46 protein was predicted by TOPCONS, TMHMM, and PROTTER, and the results indicated that this protein possessed a transmembrane region potentially spanning amino acid residues 39 to 57. Its N-terminus and C-terminus were located extracellularly and intracellularly, respectively, and exhibited characteristics similar to those of class III Holins (Fig. 1A). The Western blotting results demonstrated that Hol 46 can be recognized by anti-His-tag antibodies, with a band at 16 kDa (Fig. 1B). The concentration of the protein in the whole-cell supernatant was approximately 0.8 µg/µL according to the BCA method.

The subcellular localization of GFP-tagged Hol 46 protein (GFP-Hol 46) was characterized using fluorescence microscopy. Following IPTG induction, bacterial cells were stained with the cell membrane-specific dye FM4-64. In the control group (expressing GFP alone via the pET-28a vector), green fluorescence was diffusely distributed throughout the cytoplasm. In contrast, cells expressing GFP-Hol 46 protein exhibited fluorescence predominantly on the cell membrane, with intense GFP signals tightly overlapping with those of the FM4-64-stained membrane structures. High-resolution imaging revealed punctate colocalization patterns at the plasma membrane, indicating clustered membrane association (Fig. 1C). Fractionation analysis further demonstrated that Hol 46 was enriched in the membrane protein fraction (Fig. 1D), confirming its integral localization to the *E. coli* cell membrane and suggesting a putative membrane-associated biological role.

The MIC and MBC of Hol 46 against *A. hydrophila* Ah152 were determined to be ≥0.3 µg/µL and ≥0.6 µg/µL, respectively. After Hol 46 was expressed, the growth of the BL21 host cells was inhibited. Colony counting revealed a significant decrease in the number of pET-28a-Hol 46-BL21 cells after IPTG induction. The number of pET-28a-S105-BL21 cells also decreased compared with that in the control group (pET-28a-S105-BL21-IPTG) following the induction of S105 expression. A concentration of 0.6 µg/µL Hol 46 effectively inhibited the growth of *A. hydrophila* Ah152, decreasing the cell count to 2.6 log units after 6 h. Furthermore, the bactericidal effect of Hol 46 NC on *A. hydrophila* Ah152 was not significantly different from that of Hol 46 (Fig. 1E).

When the cell membrane was intact, cellular nucleic acids were confined to the cytosol, resulting in negligible absorbance of the extracellular solution at 260 nm. Conversely, when the membrane integrity was compromised, nucleic acids were released into the surrounding medium, leading to a measurable increase in the absorbance at this wavelength. This change in the optical density directly signified the disruption of membrane barrier function and the leakage of intracellular components, serving as a quantitative indicator of membrane permeability. Hol 46 expression also caused damage to the cell membrane of both the host BL21 strain and *A. hydrophila* Ah152, resulting in nucleic acid leakage (Fig. 1F). β-Galactosidase, which is typically an intracellular enzyme, is not normally secreted into the extracellular space; however, under conditions of cell membrane damage, some β-galactosidases can leak out of the cell. Compared with that in pET-28a-Hol 46-BL21, which was not induced by IPTG, after Hol 46 was expressed in IPTG-induced pET-28a-Hol 46-BL21, cell membrane permeability increased significantly (*P* < 0.0001), leading to elevated β-galactosidase activity owing to its leakage into the extracellular space. Similarly, *A. hydrophila* Ah152 exhibited increased cell membrane permeability and elevated β-galactosidase activity in the presence of Hol 46 (Fig. 1G). Rh123 fluorescence assays were employed to evaluate alterations in the transmembrane potential of *A. hydrophila* Ah152 and *E. coli* BL21 (DE3) expressing Hol 46 (Fig. 1H). Treatment of *A. hydrophila* Ah152 with Hol 46 resulted in a significant reduction in Rh123 fluorescence intensity (*P < 0.0001*), indicating membrane depolarization. Similarly, S105 had a comparable effect on membrane depolarization in *A. hydrophila* Ah152. IPTG-induced expression of Hol 46 in pET-28a-Hol 46-BL21 also caused a similar decrease in the fluorescence intensity (*P* < 0.0001), whereas uninduced controls maintained their baseline fluorescence. These findings demonstrated that Hol 46 disrupted the transmembrane potential in both *A. hydrophila* Ah152 and *E. coli* BL21(DE3). The membrane potential of the host bacteria was disrupted with the induction of S105 and Hol 46 NC expression. These data indicated that the Hol 46 and S105 proteins exerted the same effects on cells in terms of membrane integrity and toxicity. FACS analysis revealed that after incubation with Hol 46, 32.6% of the *A. hydrophila* Ah152 cells presented fluorescence signals from the PI dye, whereas 26.3% of IPTG-induced pET-28a-Hol 46-BL21 cells presented PI signals, both of which were greater than those observed in the noninduced group (Fig. 1I). These results demonstrate that Hol 46 induces membrane damage and increases the permeability of the cell membrane.

### The C-terminal domain of Hol 46 mediates membrane damage in bacterial cells

**Fig 2:**
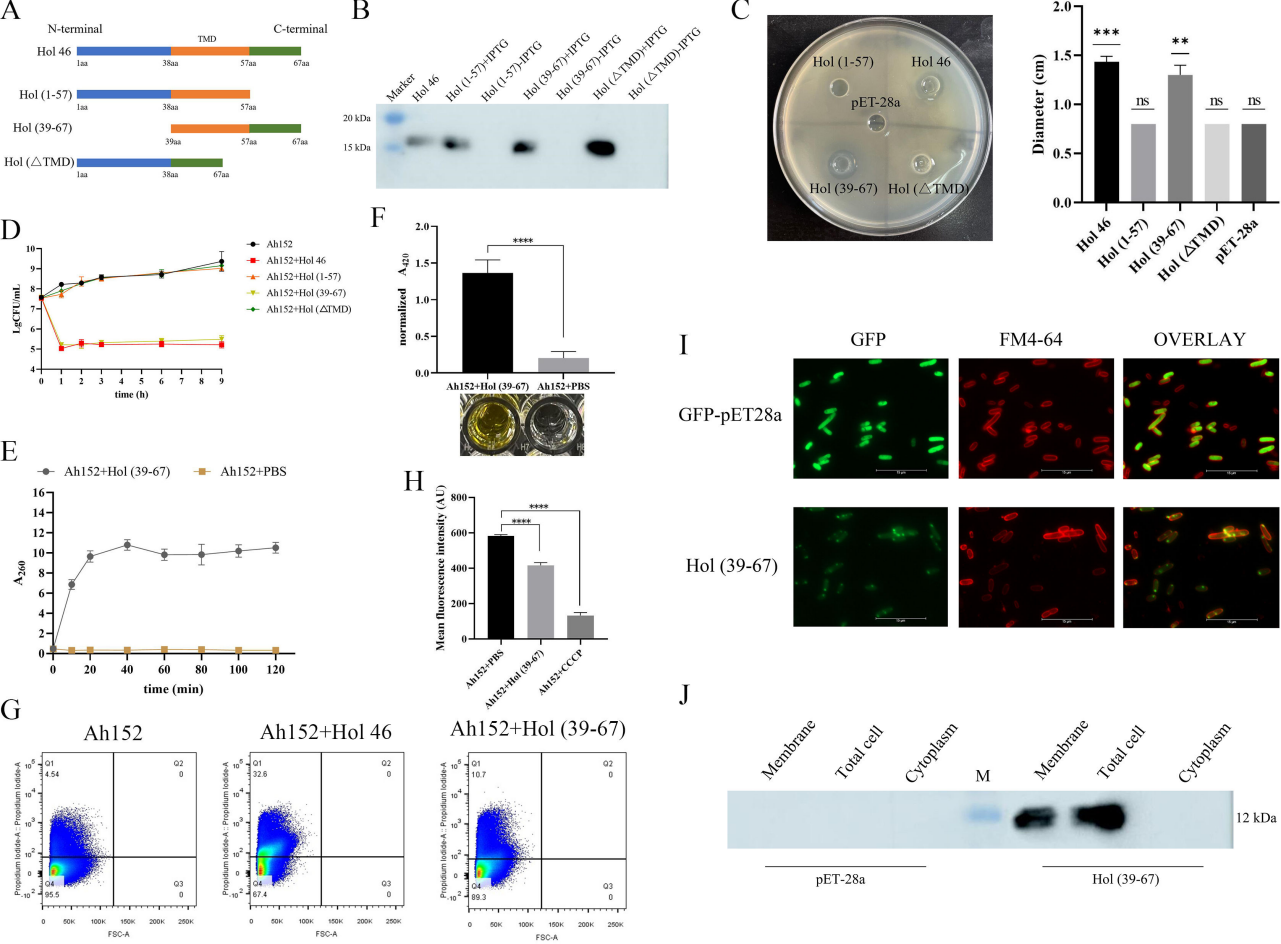
The C-terminus of Hol 46 played an important role in membrane perforation. (**A**) Schematic diagram of the design of the Hol 46 truncated proteins. (**B**) Analysis of truncated protein expression by Western blotting. (**C**) Bacteriostatic effects of the recombinant proteins Hol 46, Hol (1–57), Hol (39–67), and Hol (ΔTMD) on *A. hydrophila* Ah152 and statistical analysis of their bacteriostatic diameters. ∗*P* < 0.05, ∗∗*P* < 0.01, ∗∗∗*P* < 0.001, ∗∗∗∗*P* < 0.0001, ns indicates no significant difference. (**D**) Effects on the survival rate of *A. hydrophila* Ah152 cells. (**E**) Measurement of the ultraviolet absorption of the bacterial suspension. (**F**) Determination of β-galactosidase activity. (**G**) FACS analysis of cell membrane integrity. The images of the Ah152 group and Ah152 + Hol 46 group are identical to those in Fig. 1I and are included here as controls. (**H**) Effect of the Hol (39–67) protein on the transmembrane potential of *A. hydrophila* Ah152. (**I**) Fluorescence microscopy images of *Escherichia coli* expressing GFP-Hol (39–67) and GFP. (**J**) Subcellular localization of the protein Hol (39–67) determined by Western blotting.

On the basis of bioinformatics predictions of the transmembrane region of Hol 46, full-length Hol 46 was truncated into three proteins with different lengths: Hol (1–57), with the N-terminus containing the transmembrane region; Hol (39–67), with the C-terminus containing the transmembrane region; and Hol (ΔTMD), lacking the transmembrane region (Fig. 2A). The truncated proteins can be expressed with bands at approximately 15, 12, and 14 kDa (Fig. 2B). The antibacterial activity of each truncated protein against *A. hydrophila* Ah152 was determined via the Oxford Cup method. The antibacterial activity of the truncated proteins was similar to that of full-length Hol 46 only when both the transmembrane region and the C-terminal domain were present; Hol (1–57) and Hol (ΔTMD) did not show antibacterial activity against *A. hydrophila* Ah152. Hol 46 and Hol (39–67) significantly inhibited cell growth, whereas Hol (1–57) and Hol (ΔTMD) showed minimal growth inhibitory effects (Fig. 2C). Notably, Hol (39–67) reduced the cell count of *A. hydrophila* Ah152 and exhibited bactericidal activity similar to that of full-length Hol 46 (Fig. 2D), indicating that the C-terminal domain and transmembrane region were crucial for the antimicrobial function of this protein. These results confirmed that both Hol 46 and its truncated form Hol (39–67) effectively disrupted the *A. hydrophila* Ah152 cell membranes and inhibited its growth.

Both Hol 46 and its truncated variant Hol (39–67) disrupted the *A. hydrophila* Ah152 cell membrane (Fig. 2E). Nucleic acid leakage assays demonstrated that these proteins induced significant release of intracellular nucleic acids into the supernatant, directly reflecting compromised membrane integrity and increased membrane permeability. Furthermore, β-galactosidase activity assays, an established method to examine membrane permeability, revealed an increase in the A_420_ of the treated cells compared with control cells, corroborating the nucleic acid leakage assay data (Fig. 2F). FACS analysis validated these findings: both Hol 46 and Hol (39–67) induced a significant increase in PI fluorescence intensity, indicating enhanced membrane permeability and loss of structural integrity (Fig. 2G). Consistent with Hol 46, Hol (39–67) induced comparable transmembrane potential depolarization in *A. hydrophila* Ah152, as quantified by Rh123 fluorescence assays (Fig. 2H). Collectively, these results demonstrated that the truncated Hol (39–67) retained the membrane disruptive capacity of full-length Hol 46, mediating comparable alterations in both membrane structure and functional permeability.

The results of the cell localization experiments revealed that the fluorescence of GFP in the control group was localized within the cytoplasm, whereas the Hol (39–67) protein was localized mainly at the cell surface and colocalized with the FM4-64 dye, indicating that Hol (39–67) accumulated primarily on the cell membrane (Fig. 2I). Furthermore, after the plasmid containing Hol (39–67) was transformed into *E. coli* BL21 and its expression was induced, the Hol (39–67) protein was detected only in the whole-cell and membrane protein samples, confirming its association with the host cell membrane (Fig. 2J). These results indicated that the subcellular localization of Hol (39–67) was similar to that of full-length Hol 46, as both were expressed primarily on the cell membrane.

### Residues Glu66 and Lys63/Lys64 are critical for the membrane-damaging activity of the Hol 46 protein

**Fig 3:**
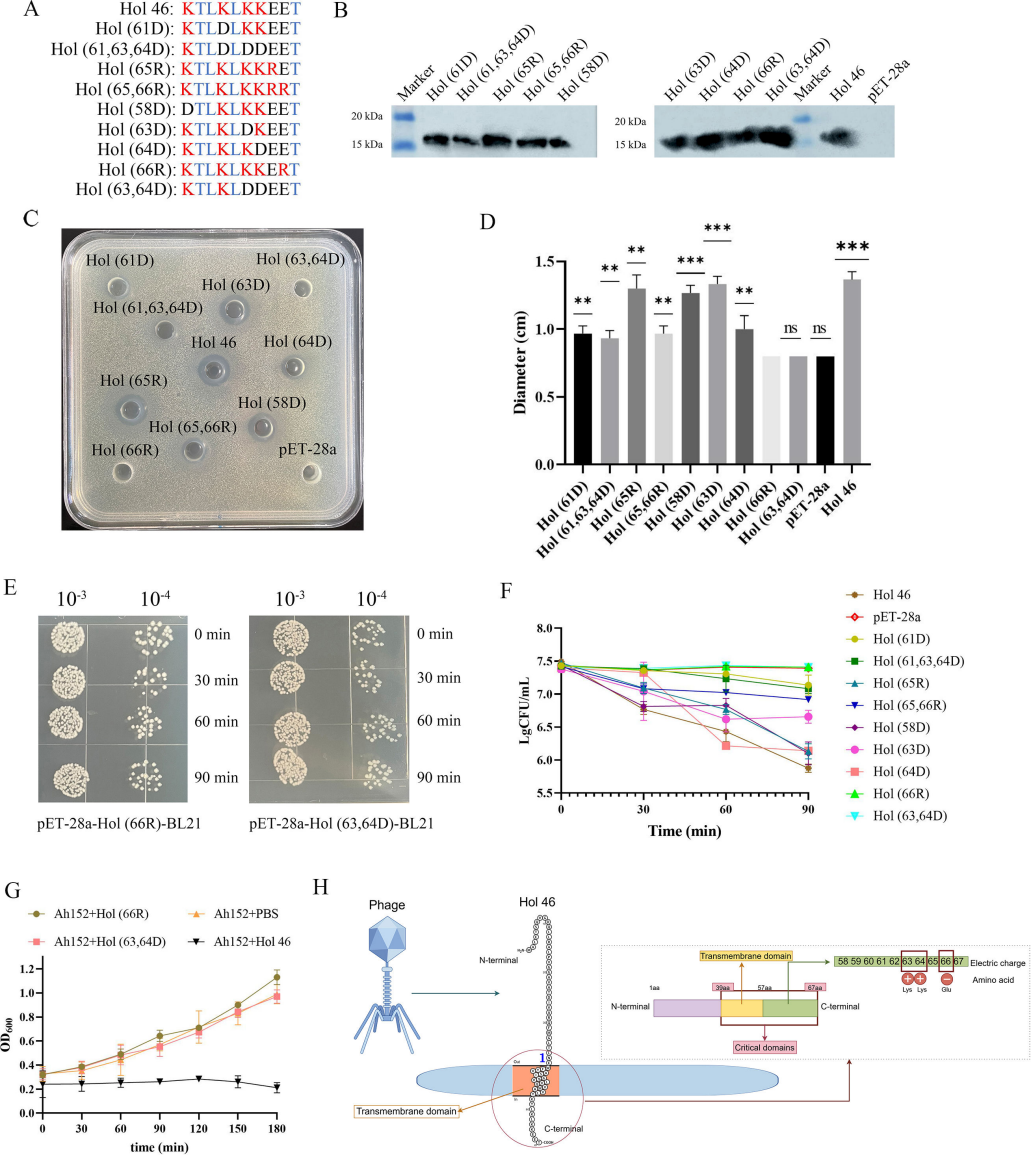
Glu at position 66 and Lys at positions 63 and 64 of the Hol 46 protein were crucial for its pore-forming activity. (**A**) Schematic diagram of the charged amino acid mutations in the C-terminal domain of Hol 46. Red represents positively charged amino acids, black represents negatively charged amino acids, and blue represents uncharged amino acids. (**B**) Analysis of mutant protein expression by immunoblotting. (**C**) Bacteriostatic effects of the recombinant proteins Hol (61D), Hol (61,63,64D), Hol (65R), Hol (65,66R), Hol (58D), Hol (63D), Hol (64D), Hol (66R), and Hol (63,64D) on *A. hydrophila* Ah152 and (**D**) statistical analysis of their bacteriostatic diameters. ∗*P* < 0.05, ∗∗*P* < 0.01, ∗∗∗*P* < 0.001, ∗∗∗∗*P* < 0.0001, ns indicates no significant difference. (**E**) BL21 cells expressing mutant proteins were counted and (**F**) analyzed at different time points. (**G**) Effects of the Hol (66R) and Hol (63,64D) proteins on *A. hydrophila* Ah152. (**H**) The membrane-disruptive activity of Hol 46 is mediated by its C-terminal domain. Glu66 and Lys63/Lys64 within the C-terminal domain work in concert with the TMD to decrease the membrane integrity.

The charge distribution of the C-terminal domain of Holin is critical for its perforin activity (31, 32). We introduced mutations into various charged amino acids at the C-terminus of Hol 46 to further investigate the influence of the C-terminus on perforin activity. We constructed and expressed the mutated proteins on the basis of the designed mutations outlined in Fig. 3A. SDS‒PAGE analysis, followed by incubation with an anti-His tag monoclonal antibody and a goat anti-mouse IgG/HRP secondary antibody, revealed that all the mutated proteins exhibited bands of approximately 16 kDa (Fig. 3B), confirming the successful expression of the mutated proteins.

BL21(DE3) strains harboring Hol 46 mutant constructs were induced with IPTG, and cell-free supernatants were collected to assess the antibacterial activity. Most mutant proteins exhibited intermediate antimicrobial efficacy, with the activity ranging between that of the positive control (full-length Hol 46) and that of the negative control (pET-28a empty vector cell-free supernatants). Notable exceptions were the Hol 46-(66R) and Hol 46-(63D/64D) mutants, whose antibacterial activity against *A. hydrophila* Ah152 was completely lost (Fig. 3C and D). These findings underscored the critical roles of the glutamic acid at position 66 and lysines at positions 63 and 64 in the perforin mechanism of Hol 46. Bacterial cells were counted for induced pET-28a-Hol 46-(66R)-BL21 and pET-28a-Hol 46-(63D/64D)-BL21 cultures. No significant differences in OD₆₀₀ values were observed between the mutant strains and the wild-type strain, confirming that the protein expression did not affect BL21 cell viability (Fig. 3E and F). We also observed that BL21 strains expressing the mutant protein displayed colonies of varying sizes. This likely reflected heterogeneity in protein expression levels due to preinduction leaky expression. Specifically, compared with larger colonies, smaller colonies may have accumulated higher levels of the protein, leading to more pronounced growth inhibition and thus slower growth. Incubation with Holin and Ah152 further demonstrated that, unlike the Hol 46 treatment group, the Hol 46-(66R) and Hol 46-(63D/64D) groups exhibited time-dependent increases in the Ah152 OD₆₀₀ values, indicating a failure to inhibit bacterial growth (Fig. 3G). These results collectively validated the essential roles of glutamic acid at position 66 and lysines at positions 63 and 64 in the antimicrobial function of Hol 46. The membrane-disruptive activity of Hol 46 was mediated by its C-terminal domain, where residues R66 and D63/D64 worked in concert with the TMD to compromise membrane integrity (Fig. 3H).

### Lys 17 exhibited bactericidal activity after the bacterial outer membrane was damaged

**Fig 4:**
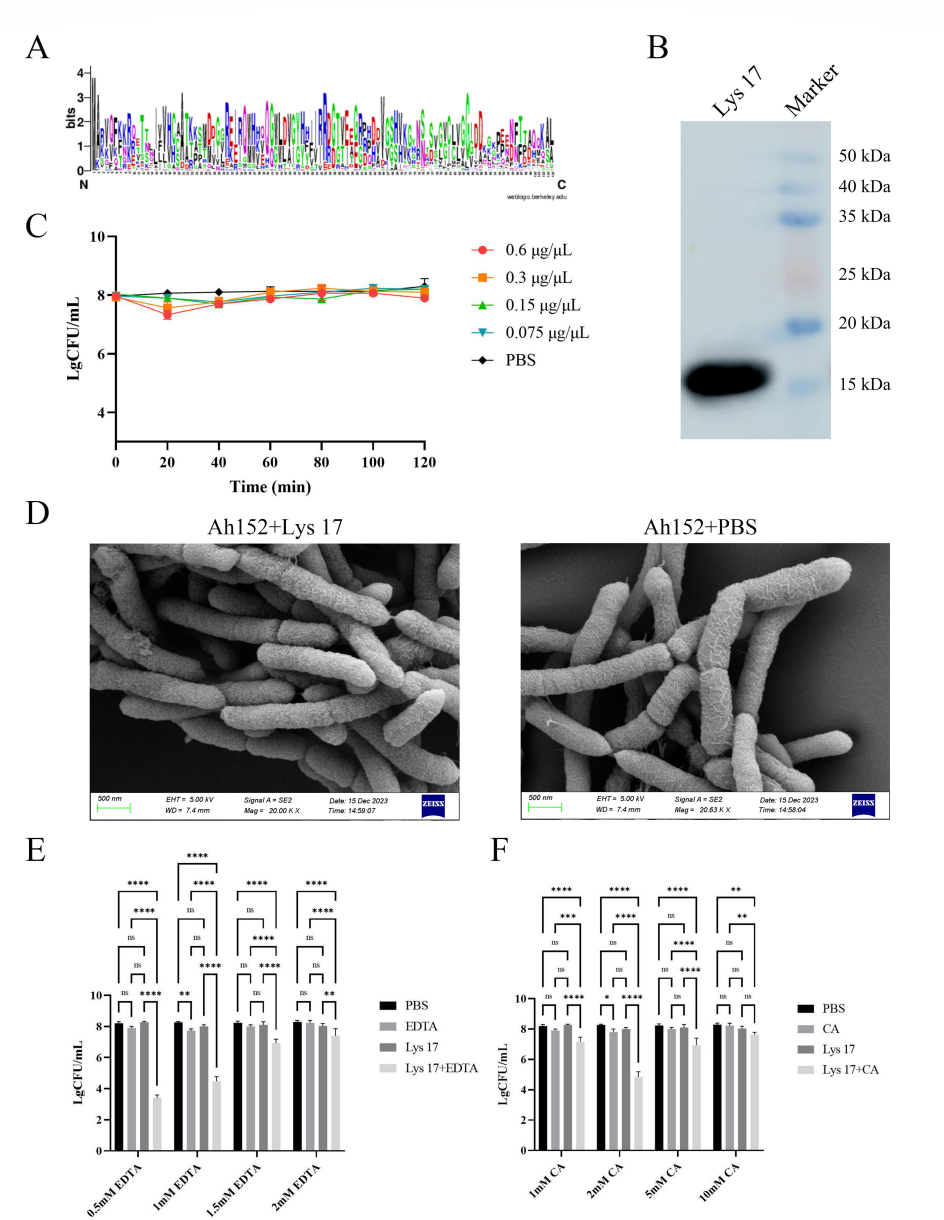
Lys 17 exhibited bacteriolytic activity after the bacterial outer membrane was damaged. (**A**) Weblogo image showing the conservation of the Lys 17 amino acid fragment. (**B**) Western blot analysis of protein expression. (**C**) The activity of the Lys 17 protein at different concentrations. (**D**) SEM images of *A. hydrophila* Ah152 cells with or without Lys 17 treatment. (**E**) Effects of different concentrations of EDTA on the function of the Lys 17 protein. ∗*P* < 0.05, ∗∗*P* < 0.01, ∗∗∗*P* < 0.001, ∗∗∗∗*P* < 0.0001, ns indicates no significant difference. (**F**) Effects of different concentrations of citric acid on the function of the Lys 17 protein.

Weblogo analysis of the Lys 17 sequence revealed a substantial number of conserved residues (Fig. 4A). Western blotting revealed a prominent band for Lys 17 at approximately 16 kDa (Fig. 4B). Following incubation with various concentrations of Lys 17, the number of *A. hydrophila* Ah152 cells in each treatment group remained stable for 120 min compared with that in the PBS control group (Fig. 4C). SEM revealed that after incubation with 0.6 µg/µL Lys 17, the cell morphology of *A. hydrophila* Ah152 did not differ from that of the control cells, suggesting that Lys 17 did not influence the activity or morphology of *A. hydrophila* Ah152 (Fig. 4D).

The outer membrane of gram-negative bacteria acts as a natural barrier, preventing phage lysins from directly exerting lytic activity (33, 34). Nevertheless, permeabilizers such as EDTA and organic acids can increase the permeability of the outer membrane, allowing phage lysins to traverse the outer membrane and contact the bacterial peptidoglycan layer, resulting in lytic activity (35). Treatment of *A. hydrophila* Ah152 with 0.5 mM EDTA and 2 mM citric acid resulted in the greatest lytic activity of Lys 17 observed. Further increases in the EDTA and citric acid concentrations did not increase the lytic activity of Lys 17 against *A. hydrophila* Ah152 (Fig. 4E and F).

### Hol 46_Lys 17 exhibited more efficient bactericidal activity

**Fig 5:**
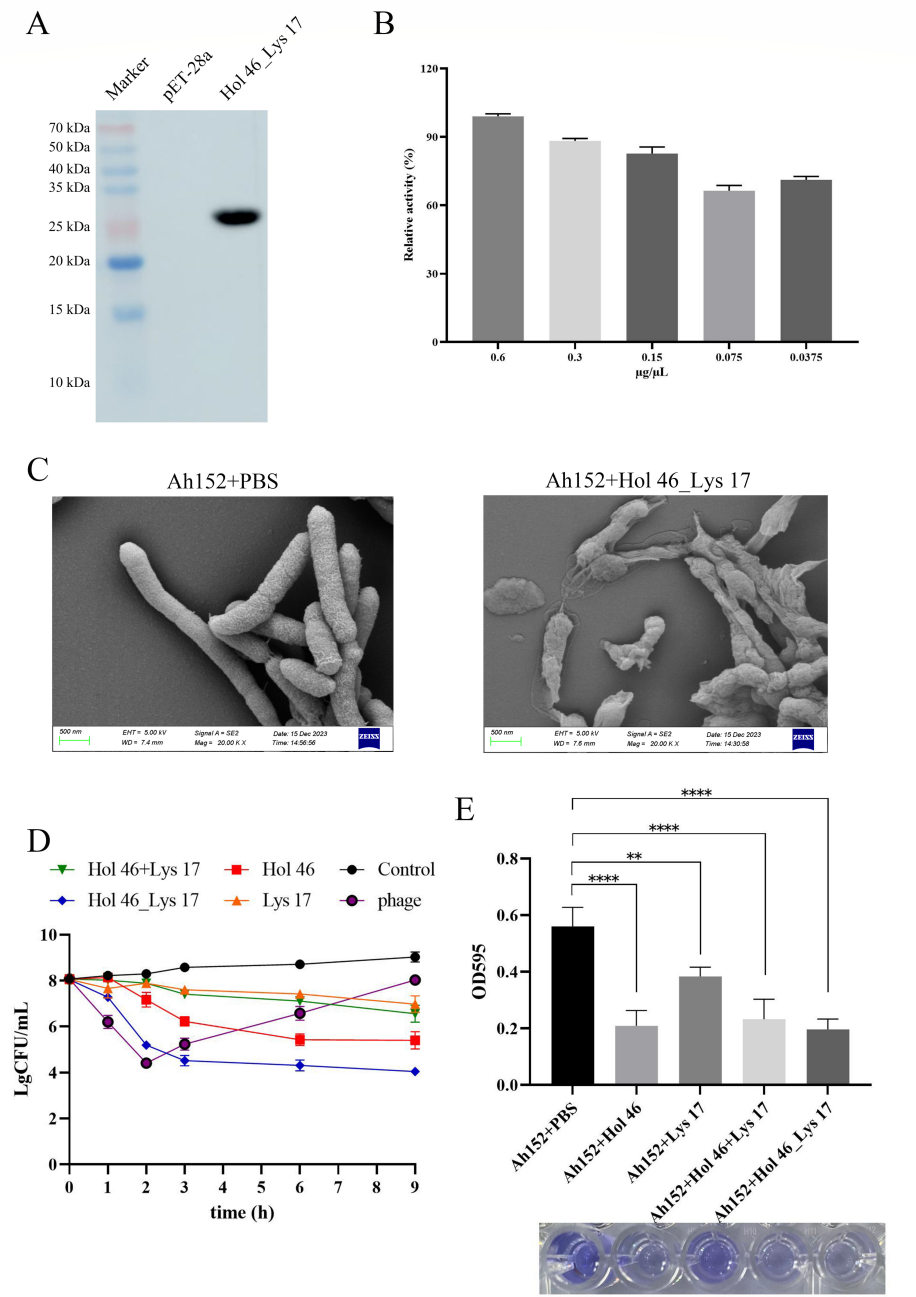
Hol 46_Lys 17 exhibited bactericidal activity. (**A**) Western blot analysis of Hol 46_Lys 17 protein expression. (**B**) Activity of the Hol 46_Lys 17 protein at different concentrations (0.6 µg/µL, 0.3 µg/µL, 0.15 µg/µL, 0.075 µg/µL, and 0.0375 µg/µL). (**C**) SEM images of *A. hydrophila* Ah152 cells treated with PBS and the Hol 46_Lys 17 protein (0.6 µg/µL) for 3 h. (**D**) Bactericidal activity of the fusion protein Hol 46_Lys 17. (**E**) Ability of the fusion protein Hol 46_Lys 17 to disrupt *A. hydrophila* Ah152 biofilms. ∗*P* < 0.05 , ∗∗*P* < 0.01, ∗∗∗*P* < 0.001, ∗∗∗∗*P* < 0.0001.

Western blotting revealed a distinct protein band corresponding to Hol 46_Lys 17 at 26 kDa (Fig. 5A). Hol 46_Lys 17 demonstrated significant bactericidal activity against *A. hydrophila* Ah152 at a concentration of 0.6 µg/µL, with its antibacterial efficacy decreasing as the concentration decreased. Even at a low concentration of 0.0375 µg/µL, more than 60% antibacterial effectiveness was retained (Fig. 5B). SEM revealed that following incubation with Hol 46_Lys 17 (0.6 μg/μL), *A. hydrophila* Ah152 cells exhibited shrinkage, dehydration, and collapse (Fig. 5C). *In vitro* bactericidal assays indicated that the use of the phage PZL-Ah152 to lyse *A. hydrophila* Ah152 led to the emergence of resistant bacteria after 2 h (Fig. 5D). A killing assay in which Hol 46 was overexpressed during the first hour of coincubation revealed no significant lytic effect on *A. hydrophila* Ah152. However, after 2 h of incubation, the Hol 46 protein exhibited a continuous inhibitory effect on bacterial growth, and the cell count decreased by 2.6 log units after 6 h. Lys 17 did not exhibit significant cytotoxicity during a 9 h incubation with *A. hydrophila* Ah152 (Fig. 5D). Although Hol 46 had perforation capabilities, a 1:1 mixture of Hol 46 and Lys 17 applied to *A. hydrophila* Ah152 failed to produce the anticipated lytic activity (Fig. 5D). Compared with the other groups, Hol 46_Lys 17 reduced *A. hydrophila* Ah152 counts by 4 log units during 9 h of incubation, demonstrating sustained lytic activity and a reduced propensity for inducing bacterial resistance (Fig. 5D). Furthermore, Hol 46_Lys 17 exhibited a broader host range, displaying lytic activity against strains of *E. coli* (3/12) and *Salmonella* (5/29) (Table S3). In natural environments, *A. hydrophila* predominantly exists in biofilms, which exhibit greater resistance and survival capabilities than planktonic strains do. We demonstrated that the protein Hol 46_Lys 17 can significantly degrade biofilms (*P* < 0.0001) (Fig. 5E).

### Effects of Hol 46_Lys 17 on the transcriptome of *A. hydrophila* Ah152

**Fig 6:**
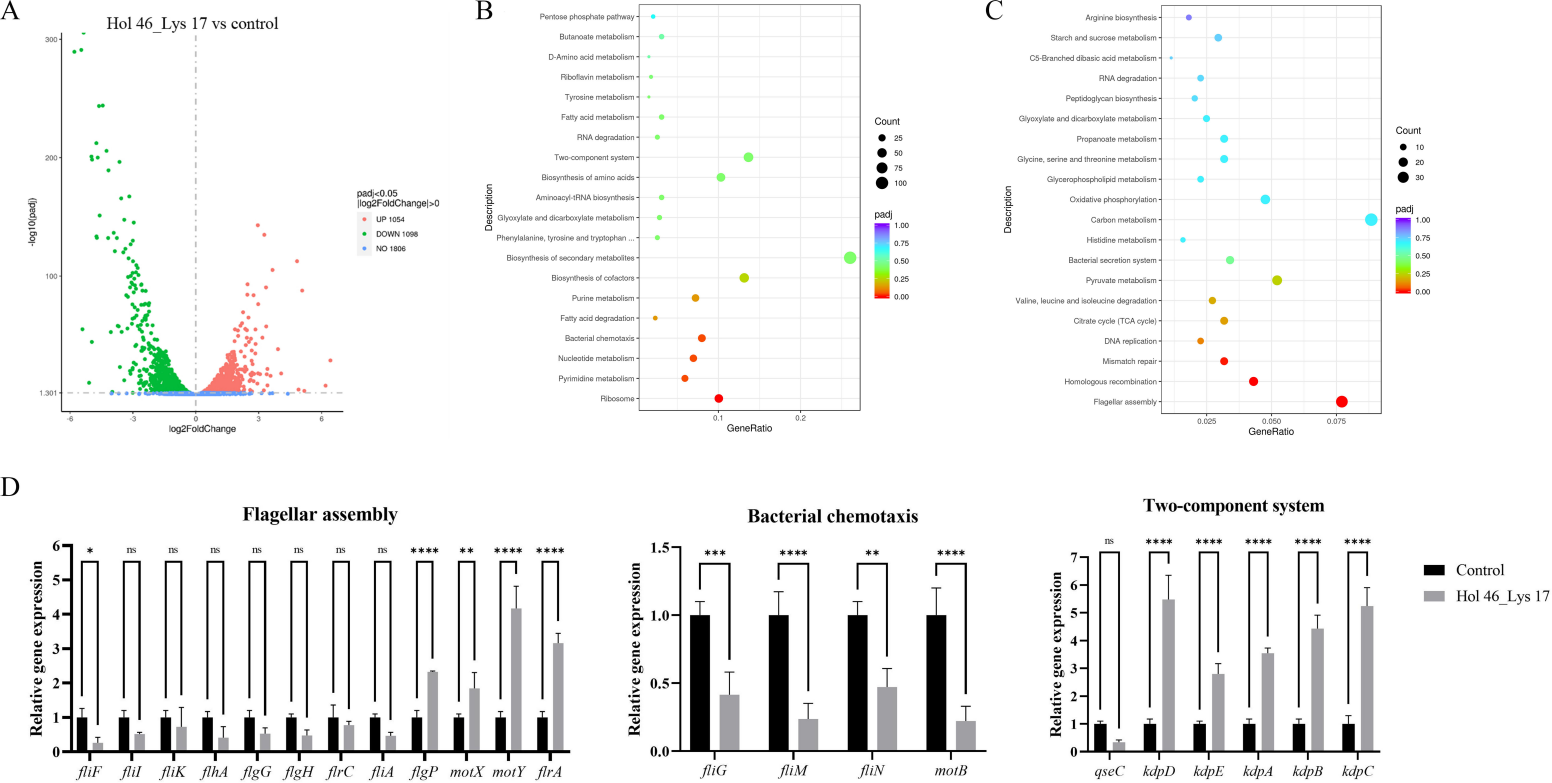
Effects of Hol 46_Lys 17 on the transcriptome of *A. hydrophila* Ah152. (**A**) Volcano plot analysis. (**B**) KEGG enrichment analysis of upregulated DEGs after Hol 46_Lys 17 treatment vs control treatment. Statistical significance was determined using adjusted *P* values. Data points are colored according to their Benjamini-Hochberg-corrected *P* values (padj), with lower padj values (padj < 0.05) indicating greater significance. (**C**) KEGG enrichment analysis of downregulated DEGs after Hol 46_Lys 17 treatment vs control treatment. (**D**) The relative expression levels of genes related to flagella, bacterial chemotaxis, and the TCS were analyzed via RT-qPCR. ∗*P* < 0.05, ∗∗*P* < 0.01, ∗∗∗*P* < 0.001, ∗∗∗∗*P* < 0.0001, ns indicates no significant difference.

Compared with no treatment, treatment with *A. hydrophila* Ah152 with 5 µg/mL Hol 46_Lys 17 resulted in 80% bacterial survival. On the basis of this dose-response relationship, transcriptomic analysis was performed on *A. hydrophila* Ah152 cultures incubated with 5 µg/mL Hol 46_Lys 17 to investigate the sublethal effects while maintaining sufficient viability to preserve RNA integrity. Transcriptomic analysis was used to compare genes that were differentially expressed between *A. hydrophila* Ah152 and *A. hydrophila* Ah152 treated with Hol 46_Lys 17 (Fig. 6A). RT-qPCR analysis (using the primers listed in Table S4) revealed that Hol 46_Lys 17 affected gene expression in *A. hydrophila* Ah152, particularly genes associated with the regulation of flagellar synthesis, bacterial chemotaxis, and two-component systems (TCSs) (Fig. 6D). In the flagellar synthesis pathway, 34 differentially expressed genes (DEGs) related to flagellum composition tended to be downregulated and were directly associated with flagellum assembly. Furthermore, in the bacterial chemotaxis pathway, 15 DEGs linked to flagella were also downregulated. Analysis of the TCS likewise revealed the downregulation of genes associated with flagellum formation and the upregulation of genes related to potassium ion limitation, which may further affect internal and external cellular signal transduction and adaptability.

### Hol 46_Lys 17 demonstrated therapeutic efficacy in crucian carp

**Fig 7:**
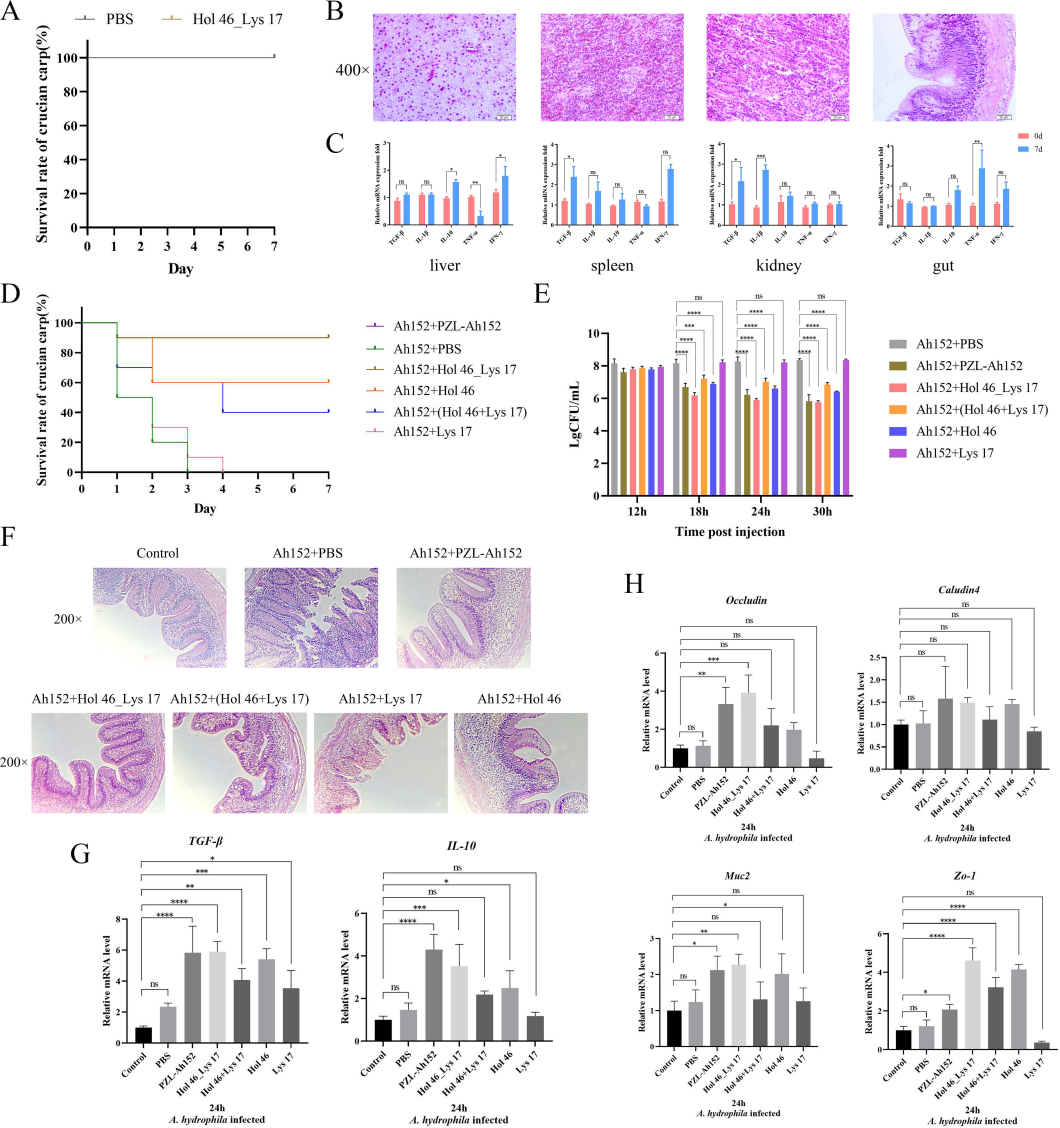
Hol 46_Lys 17 demonstrated therapeutic efficacy in crucian carp. (**A**) Survival rates of crucian carp over 7 days after IP injection of PBS or the Hol 46_Lys 17 protein. (**B**) Evaluation of the safety of the Hol 46_Lys 17 protein after IP injection into crucian carp. On day 7, various tissues were collected and stained with H&E to observe pathological changes. (**C**) Changes in the transcription of specific genes in the liver, spleen, kidney, and gut of crucian carp 7 days after IP injection of the Hol 46_Lys 17 protein.∗*P* < 0.05, ∗∗*P* < 0.01, ∗∗∗*P* < 0.001, ∗∗∗∗*P* < 0.0001, ns indicates no significant difference. (**D**) Effects of each protein mixture on the survival rate of infected crucian carp. (**E**) Effects of each protein mixture on the bacterial load in infected crucian carp. (**F**) Histopathological images of the intestinal tracts from crucian carp. (**G**) mRNA expression levels of *TGF-β* and *IL-10* in the intestines of crucian carp 24 h after *A. hydrophila* infection. (**H**) mRNA expression levels of *Occludin, Claudin4*, *Muc2,* and *Zo-1* in the intestines of crucian carp 24 h after *A. hydrophila* infection.

The safety of Hol 46_Lys 17 as a potential therapeutic agent was tested by IP injection of Hol 46_Lys 17 into crucian carp. In the safety test, the survival rate of crucian carp was 100% (Fig. 7A). In addition, the tissues collected from crucian carp treated with Hol 46_Lys 17 showed no pathological changes, as shown in Fig. 7B. The mRNA expression levels of *TGF-β*, *IL-1β*, *TNF-α*, *IFN-γ*, and *IL-10* were quantified via qPCR. Compared with that at 0 days, the transcript levels of *TGF-β* mRNA in the spleen and kidney increased on the seventh day (Fig. 7C). In the intestines, the transcript levels of *IL-10*, *TNF-α*, and *IFN-γ* also began to increase on the seventh day (Fig. 7C).

The MLD of *A. hydrophila*-Ah152 to carp was 10^8^ CFU, and the effects of PZL-Ah152, Hol 46_Lys 17, Hol 46, Lys 17, and Hol 46 + Lys 17 on *A. hydrophila*-infected crucian carp were evaluated. Endotoxin assays conducted with various proteins prior to treatment yielded results below 1 EU/mg, indicating a favorable safety profile. One hour after the intraperitoneal injection of a 2 × MLD dose of *A. hydrophila* into crucian carp, several proteins, including phages, were administered for treatment. In the control group (PBS), all the fish died within 3 days, whereas the Lys 17 treatment group experienced total mortality within 4 days. The Hol 46 protein-treated group presented a 60% survival rate after 7 days; however, the combination of Hol 46 and Lys 17 did not increase survival (40% survival over the same period). Notably, the treatment efficacy of Hol 46_Lys 17 paralleled that of phage PZL-Ah152, with a survival rate of 90% after 7 days (Fig. 7D). Furthermore, the bacterial loads in the crucian carp were assessed at various time points. The bacterial load in the PBS-treated control group remained at 1 × 10^8^ CFU/mL, whereas treatment with the phage PZL-Ah152 or Hol 46_Lys 17 significantly reduced the load of *A. hydrophila* Ah152 in the intestines within 18 h after infection (*P* < 0.0001), with continued suppression of bacterial growth. In contrast, treatment with a mixture of the Hol 46 and Lys 17 proteins or Lys 17 alone did not substantially alleviate the bacterial burden (Fig. 7E). These data suggested that Hol 46_Lys 17 could effectively treat *A. hydrophila* infection.

Pathological changes in the intestines of crucian carp in each treatment group were assessed via H&E staining. In the control and Lys 17 treatment groups, the intestinal villi exhibited epithelial cell dissolution, necrosis, or the dissolution of lamina propria cells and compromised mucosal integrity, resulting in intestinal crypt loss and inflammatory cell infiltration. Conversely, the treatment groups receiving the phage PZL-Ah152 and Hol 46_Lys 17 presented a progressive reduction in intestinal inflammation, reduced damage to the intestinal villi, distinct intestinal crypts, and increased goblet cell production, indicating gradual restoration of the normal intestinal architecture (Fig. 7F). The qPCR results confirmed that Hol 46_Lys 17 and phage PZL-Ah152 significantly upregulated the expression levels of anti-inflammatory and immune regulatory factors, including *TGF-β* and *IL-10,* within 24 h posttreatment (*P* < 0.001) (Fig. 7G). Additionally, Hol 46_Lys 17 treatment significantly increased the expressions of intestinal tight junction proteins (*P* < 0.01), such as *Occludin*, *Muc2*, and *Zo-1*, which are essential for maintaining the integrity and functionality of the intestinal barrier (Fig. 7H).

## DISCUSSION

The prevalence of multidrug-resistant bacteria not only promotes the spread of resistance genes and reduces the efficacy of traditional antibiotics but also poses a significant risk to the environment and public health. Bacteriophages, which are abundantly distributed in nature, possess the ability to specifically target and lyse pathogenic bacteria. Phages are gradually being accepted as alternatives/complements to antibiotic therapy and have been successfully used against many bacterial pathogens that cause diseases in animals and humans.

The majority of phages (approximately 95%) utilize double-stranded DNA (dsDNA) as their genetic material, with genome sizes typically ranging from 15 to 500 kb. These dsDNA phages commonly employ a two-component “holin–lysin” system to lyse host bacterial cells (36, 37). This efficient and specific Holin-lysin lysis system involves the synthesis of Holin during the later stages of phage infection (38). Holin first forms homooligomers within the cell membrane, creating transmembrane pores at designated time points. This process allows lysins to disrupt the bacterial cell wall, resulting in lysis of the host cell and the subsequent release of progeny phages, thereby completing the infection cycle. Beyond pore formation, Holin also acts as a molecular “timer” that initiates bacterial lysis, ensuring precise temporal control over host cell disruption (39). The expression of Holin proteins via genetic engineering techniques enables the autonomous induction of bacterial lysis in hosts (40), providing novel insights into the functional mechanisms and potential applications of Holin. In contrast to the canonical Holin-lysin cooperative system employed during natural phage infection, recombinant Holin alone can form functional transmembrane pores in bacterial membranes. It has been proposed that these rafts are permeable to protons or ions across the bilayer, leading to local depolarization of the membrane, which in turn causes conformational changes that allow the packed holins to reorganize into the final pores (41). Consistent with this model, Hol 46 in our study caused disruption of membrane potential and loss of membrane integrity, culminating in cell lysis (42, 43). Govind et al. stained cells with SYTO and PI to assess the integrity of the cytoplasmic membrane in endolysin-deficient cells. They reported that before thermoinduction, the lysogens appeared to have intact cell membranes, but the membrane integrity of the S105-expressing *E. coli* λ population decreased nearly 30 min after thermoinduction (44). This pattern of membrane damage by S105 is consistent with the cell membrane impairment induced by Hol 46 in our study. Both BL21 and AH152 strains exhibited similar changes in membrane potential and growth inhibition following S105 treatment, similar to the effects observed with Hol 46. Given that a His-tag might affect the function of membrane proteins, we expressed the protein with the pET-28a empty vector (containing only the His-tag sequence), which had no significant effect on bacterial growth or membrane integrity, eliminating the potential toxicity caused by the tag itself. To confirm the effect of the position of the tag, we constructed two recombinant plasmids, one with deletion of the tag after the target gene sequence that retained the original His-tag of pET-28a vector at the N-terminus and the other with an additional His-tag at the C-terminus (dual *N* + C His-tags), and protein expression was performed. The results of functional experiments revealed no significant difference in the bacterial toxicity, including its effects on membrane potential and growth inhibitory ability, of the two tagged constructs. These results demonstrated that the location of the His-tag (N-terminus or C-terminus) did not affect the biological activity of Hol 46. Notably, studying the mechanism of action of the recombinant Holin protein is essential for understanding the bacterial lysis it mediates.

In contrast to class I and class II holins, class III holins have garnered increasing attention because of their distinct structural architectures. The Gp5 protein from the *Mycobacterium* phage Ms6 is a Holin protein characterized by a single TMD with an N-terminal, C-terminal topology (45). Bioinformatics analysis in our study indicated that Hol 46 also possessed a single TMD with a similar topology and was capable of perforating the bacterial membrane. The subcellular localization of proteins is crucial for elucidating their structure and function (46). The S^21^68 protein encoded by the Holin gene S21 of phage 21 tends to form numerous aggregates; however, upon deletion of its TMD, its distribution within the inner membrane is restricted (47). To determine the cellular localization of the Hol 46 protein, we employed GFP as a fluorescent reporter. The fluorescence tagging and membrane protein extraction assay results confirmed that Hol 46 localized to the cell membrane. Previous studies have shown that pores formed by Holins are large enough to permit the release of cytoplasmic components—such as β-galactosidase—into the culture medium (48). The presence of cytoplasmic enzymes in the culture medium served as a direct indicator of cell membrane damage. By measuring the leakage of intracellular nucleic acids, β-galactosidase activity, and the uptake of PI by bacterial cells, we validated that Hol 46 altered the bacterial cell membrane permeability and confirmed that Hol 46 can cause the cell membrane to lose its integrity. Disruption of the bacterial transmembrane potential, which is likely attributable to membrane damage (49, 50), is mediated by the Hol 46 protein through the compromise of membrane integrity via depolarization and enhanced permeability, which induces the leakage of intracellular DNA and proteins, ultimately resulting in bacterial cell death (51).

To identify the key functional regions of Hol 46, we constructed and expressed a series of truncated variants with differing amino acid lengths. The results indicated that in the presence of Hol (39–67), the growth of *A. hydrophila* Ah152 was significantly inhibited, whereas no inhibitory effect on bacterial growth was observed upon expression of Hol (1–57) or Hol (ΔTMD). These findings demonstrated that the C-terminal region of Hol 46, in conjunction with its TMD, was essential for inducing cell membrane damage. The observed reduction in cell viability, combined with decreased activity of β-galactosidase in the culture supernatant, demonstrated that the absence of the N-terminal region did not abrogate the protein’s ability to damage the membrane. Studies have shown that the highly charged C-terminal region is crucial for pore formation. The C-terminal domains of Holins are highly hydrophilic and contain clusters of consecutive basic and acidic residues, resulting in a net positive charge (31). The C-terminal domain of λS is located in the cytoplasm, and mutation of its C-terminal region demonstrated that retaining at least one basic amino acid residue at the C-terminus was sufficient for perforation to occur (31). λ Holin requires at least one positively charged amino acid to function (31, 52). In this study, the C-terminus of Hol 46 featured two consecutive positively charged lysines and one negatively charged glutamic acid, yielding an overall positive net charge. Mutation of charged amino acids at the C-terminus revealed that changing the negatively charged glutamic acid at position 66 to positively charged arginine completely abolished the activity of the mutant protein Hol (66R) against *A. hydrophila* Ah152, despite the overall net charge of the C-terminus remaining positive. Conversely, mutating the lysine residues at positions 63 and 64 with positive charges to negatively charged aspartic acid residues rendered the mutant protein Hol (63D,64D) completely inactive. This mutant lacked the cell membrane-damaging ability of Hol 46 and created a net negative charge at the C-terminus. These results suggest that the overall net charge of the C-terminus of Hol 46 does not impact its ability to damage the cell membrane; however, the negatively charged glutamic acid at position 66 and the positively charged lysines at positions 63 and 64 are essential for Hol 46 to cause cell membrane damage.

Phage lysin infection of gram-positive bacteria has been extensively studied (53). As highly promising alternatives to antibiotics, the efficiency, broad-spectrum lytic activity, and stability of these lysins against various physical and chemical factors are prerequisites and foundations for their practical application. The presence of an outer membrane in gram-negative bacteria protects their peptidoglycan layer from hydrolysis by exogenous lytic enzymes (54, 55). The phage lysin Lys 17 did not exhibit lytic activity against *A. hydrophila* Ah152, possibly because of its inability to penetrate the *Aeromonas* outer membrane. Phage lysins must be able to breach the outer membrane barrier to exert lytic activity as this allows the lysin protein to access the peptidoglycan layer (56, 57). The phage lysins Ts2631, Lyspep3, SPN9CC, Ph2119, and Lys68 can penetrate bacterial outer membranes and exhibit lytic activity (58–62). EDTA and organic acids are widely studied outer membrane permeabilizers used to increase the activity of bacterial cell wall hydrolases by promoting their penetration through the bacterial outer membrane (63–65). To improve the penetration of Lys 17 through the outer membrane of gram-negative bacteria, we added different concentrations of EDTA and citric acid for coincubation with *A. hydrophila* Ah152 in an attempt to disrupt the outer bacterial membrane. The results indicated that 0.5 mM EDTA and 2 mM citric acid effectively disrupted the outer membrane of *A. hydrophila* Ah152 without affecting bacterial counts, allowing Lys 17 to act on the bacterial peptidoglycan layer and exert a lytic effect.

The lysis systems of most gram-negative bacteriophages require both Holin and lysin, which specifically target the cytoplasmic membrane and peptidoglycan, respectively (66, 67). However, some lysis systems incorporate a third class of lytic proteins called spanins, which attack the outer membrane (68). In the present study, only the Holin-lysin system was identified in the phage PZL-Ah152, and no spanin was detected. These findings align with the results of Kongari et al., who revealed that approximately 15% of bacteriophages lack spanin-encoding genes (69). In this work, Hol 46 was fused with Lys 17, and the data indicated that the bactericidal activity of Hol 46_Lys 17 was significantly greater than that of Hol 46 alone and the Hol 46 + Lys 17 protein mixture. This enhanced activity may result from the spatial proximity of the fusion of the proteins’ active sites or their novel spatial arrangement, which enables synergistic effects that strengthen their lytic capabilities (70). Host-spectrum tests revealed that Hol 46_Lys 17 exhibited a broader host spectrum, demonstrating lytic activity not only against *Aeromonas* but also against some strains of *E. coli* (3/12) and *Salmonella* (5/29). Furthermore, compared with the control group, the Hol 46_Lys 17 group presented significantly improved biofilm degradation activity.

Transcriptomic analysis revealed that Hol 46_Lys 17 downregulated the transcription of key genes involved in flagellar assembly (e.g., *flgG* and *flgH*) and chemotaxis (e.g., *cheW, cheA,* and *motA/motB*). FlgG is a bacterial flagellar rod protein and constructs the distal rod that connects to the hook (71). FlgH (the L-ring subunit of the flagellar basal body) is a lipoprotein whose modification is important for L-ring assembly (72). The inhibition of the expression of these genes may damage the mechanical stability of flagella, resulting in incomplete assembly or a fragile structure. Chemoreceptor signaling core complexes, consisting of CheA, CheW, and methyl-accepting chemotaxis proteins (MCPs), modulate the switching of bacterial flagellar rotation, which drives cell motility (73). Additionally, MotA and MotB form the stator complex of the flagellar motor, which converts the transmembrane proton motive force into rotational torque (74, 75). Downregulation of *motA* and *motB* may therefore impair torque generation, consistent with studies in *Salmonella* showing that MotA/MotB mutations reduce motor speed (76). The KdpATPase complex, encoded by the *kdpABC* operon, is an inducible, high-affinity potassium ion transport system (77, 78). *kdpABC* encodes a potassium transport channel that is required for ATP homeostasis. K^+^ is essential for many cellular functions, including the maintenance of the intracellular pH and transmembrane potential (79). In our study, we observed an upregulation of the *kdpABC* gene within the TCS, suggesting a potential disturbance in the potassium ion transport channel. This dysregulation could further affect the transmembrane potential (80).

As a novel antimicrobial treatment strategy, phage therapy has considerable potential for high efficacy and specificity. *In vivo*, Hol 46_Lys 17 significantly reduced the *A. hydrophila* Ah152 load in the intestines of crucian carp and alleviated damage to the intestinal villi, thereby substantially improving the survival rate of crucian carp following *A. hydrophila* Ah152 infection. The differential cytokine expression profiles in the immune organs of crucian carp following IP protein administration revealed a tissue-specific immunoregulatory network with complex functional hierarchies. Although fish and mammals exhibit lineage-specific adaptations, core components of innate immunity and barrier defenses remain evolutionarily conserved. For instance, the Toll-like receptor (TLR) family is highly conserved, with most mammalian TLRs showing homology to those in fish (81). Functional conservation of TLR signaling has been validated in zebrafish and rainbow trout (82, 83). In terms of mucosal barriers, the fish gut mucosa exhibits functional parallels with mammalian epithelial barriers, utilizing mucus layers enriched in antimicrobial peptides and secretory IgA analogs (84, 85). These evolutionarily conserved mechanisms support the cautious extrapolation of findings from mammalian studies to fish when direct data on fish are limited. In the spleen and kidney, the coordinated upregulation of TGF-β and IL-1β suggested a dynamic balance between innate immune activation and immunosuppressive pathways. On the one hand, the prototypical proinflammatory cytokine IL-1β mediates classical inflammatory cascades by activating NF-κB signaling and promoting immune cell recruitment (86). Conversely, TGF-β functions as a central immunomodulator, directly inhibiting T-cell clonal expansion, dampening the proinflammatory polarization of macrophages, and regulating dendritic cell maturation to maintain immune homeostasis (87). In the liver, the pronounced upregulation of IL-10 highlighted a conserved anti-inflammatory axis. As a master regulator of immune resolution, IL-10 restricts excessive inflammation by decreasing the production of proinflammatory cytokines (e.g., TNF-α and IL-6) and inhibiting the activation of macrophages and dendritic cells (88). Moreover, the expression of TNF-α, a pivotal effector of both proinflammatory and tissue protective responses, exhibits differential regulation across tissues. In the intestine, TNF-α facilitates epithelial barrier maintenance by inducing tight junction protein redistribution, thereby enhancing intercellular adhesion and mucosal integrity—a critical process for resistance to pathogen translocation (89).

Bacteriophage-derived proteins have been successfully employed in the treatment of infections caused by pathogens such as *Staphylococcus aureus* and *Pseudomonas aeruginosa* (90, 91). Among the key immune modulators, TGF-β and IL-10 play central roles in regulating immune responses, maintaining homeostasis, and suppressing excessive inflammation (92, 93). In the present study, RT-qPCR analysis revealed that the application of Hol 46_Lys 17 significantly upregulated the mRNA transcript levels of *TGF-β* and *IL-10* in the intestines of crucian carp. The increases in *TGF-β* and *IL-10* levels can inhibit the release of inflammatory mediators, reduce tissue inflammatory responses, and protect tissues from inflammatory damage (94). Furthermore, the upregulation of *TGF-β* and *IL-10* is essential for wound healing and tissue regeneration, facilitating the repair and recovery of damaged tissues (95). Tight junction proteins are crucial determinants of barrier function between epithelial and endothelial cells (96, 97). In this study, treatment with Hol 46_Lys 17 significantly increased the expression of tight junction-related molecules, such as *Zo-1*, *Occludin*, *Claudin4*, and *Muc2*, in the intestinal epithelium, indicating an improvement in intestinal barrier function.

### Conclusion

Currently, bacteriophages and their protein derivatives remain largely in the research and clinical trial phases; however, they exhibit considerable potential to provide innovative approaches for controlling and treating infections caused by drug-resistant bacteria. These findings underscore the necessity of developing phage-based antibacterial interventions as sustainable solutions to combat these resistant strains. Future efforts should focus on developing next-generation phage products with broadened antibacterial spectra, particularly targeting environmentally prevalent antibiotic-resistant pathogens. This strategic focus will not only enhance the applicability of phage therapy in complex ecological niches but also solidify its role as a critical intervention for maintaining environmental microbial balance and safeguarding public health from resistant infection-causing pathogens.

## Data Availability

Sequence data that support the findings of this study have been deposited in the NCBI with the primary accession code MW671054.1. The original data can be provided upon request to the corresponding author, Lei Zhang (zhanglei0221@jlau.edu.cn).

## SUPPLEMENTAL MATERIAL

Figure S1 jvi.00832-25-s0001.docx Uncut WB image.

Table S1 jvi.00832-25-s0002.docx Primers for protein construction.

Table S2 jvi.00832-25-s0003.docx Sequences of primers used for real-time quantitative PCR.

Table S3 jvi.00832-25-s0004.docx Evaluation of the bactericidal activity of fusion protein Hol 46_Lys 17.

Table S4 jvi.00832-25-s0005.docx RT-qPCR primers for transcriptome sequencing.

ASM does not own the copyrights to Supplemental Material that may be linked to, or accessed through, an article. The authors have granted ASM a non-exclusive, world-wide license to publish the Supplemental Material files. Please contact the corresponding author directly for reuse.
