# Supplementary material for: Identification and analysis of the crucial holin domain and sites and the bactericidal activity of a holin–endolysin lysis cassette from phage PZL-Ah152 against Aeromonas hydrophila
Source: J Virol. 2025 Dec 15;100(1):e00832-25. doi: 10.1128/jvi.00832-25 (PMC12817945; doi:10.1128/jvi.00832-25)

**Supplementary Figure 1. Uncut WB image**

1. **WB image of Hol 46 protein**


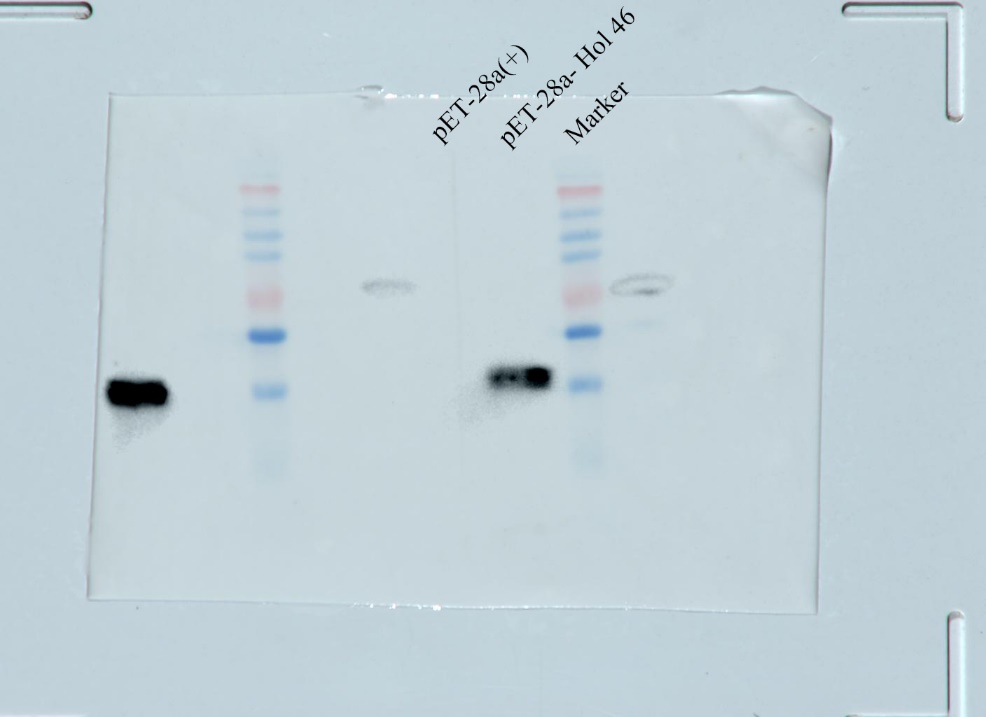


1. **WB image of Hol 46 membrane protein**


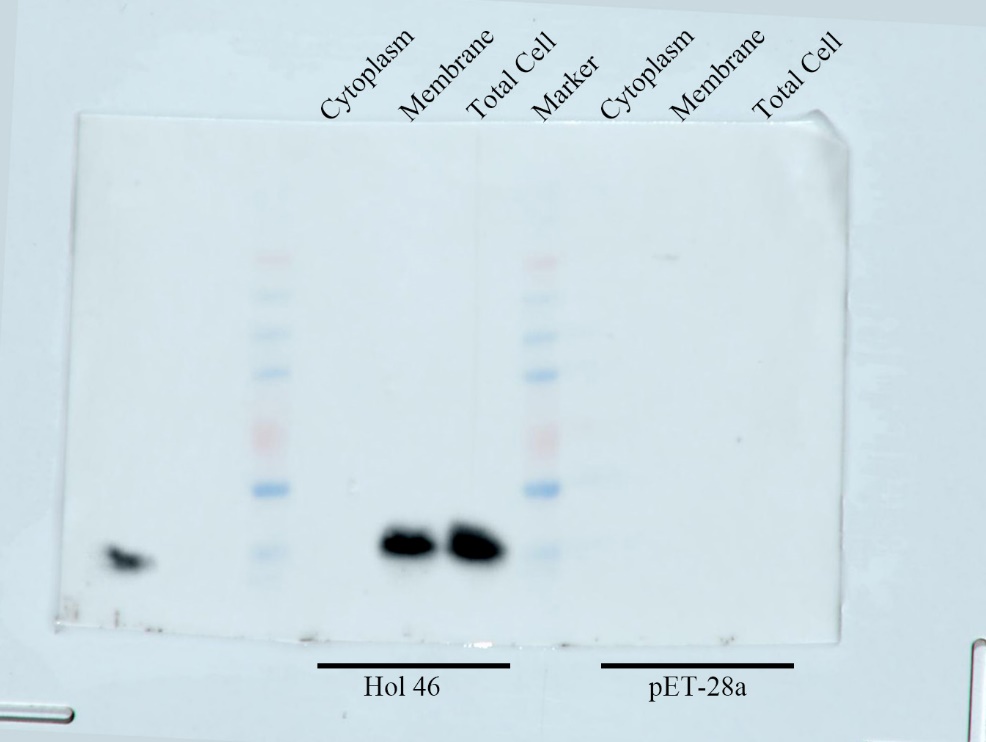


1. **WB image of Hol (1-57), Hol (39-67), and Hol (△TMD).**


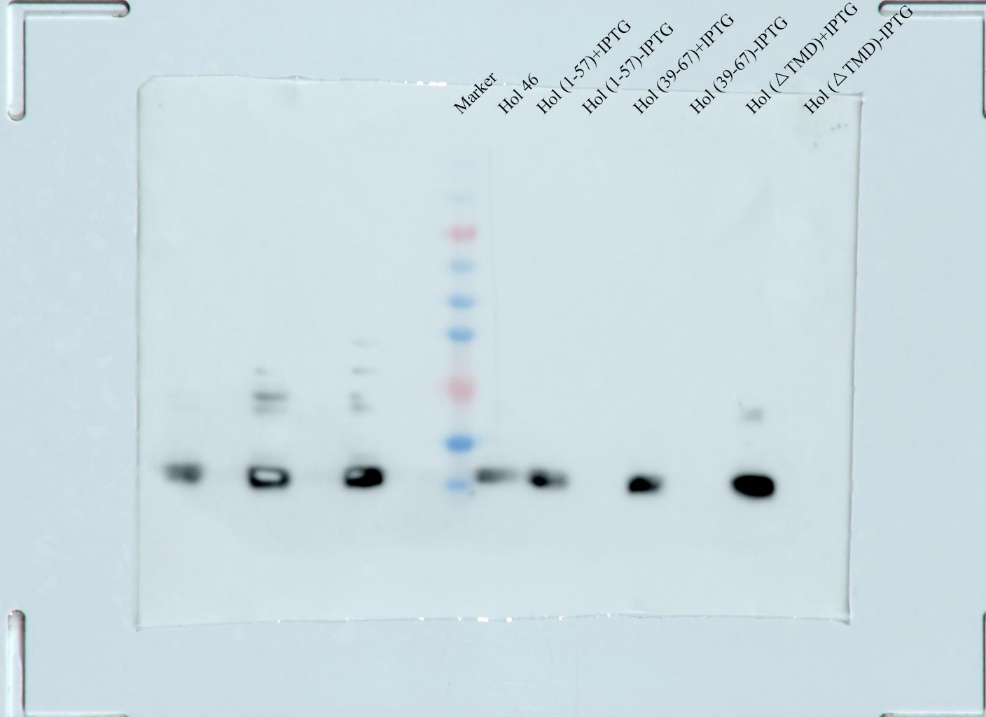


1. **WB image of Hol (39-67) membrane protein**


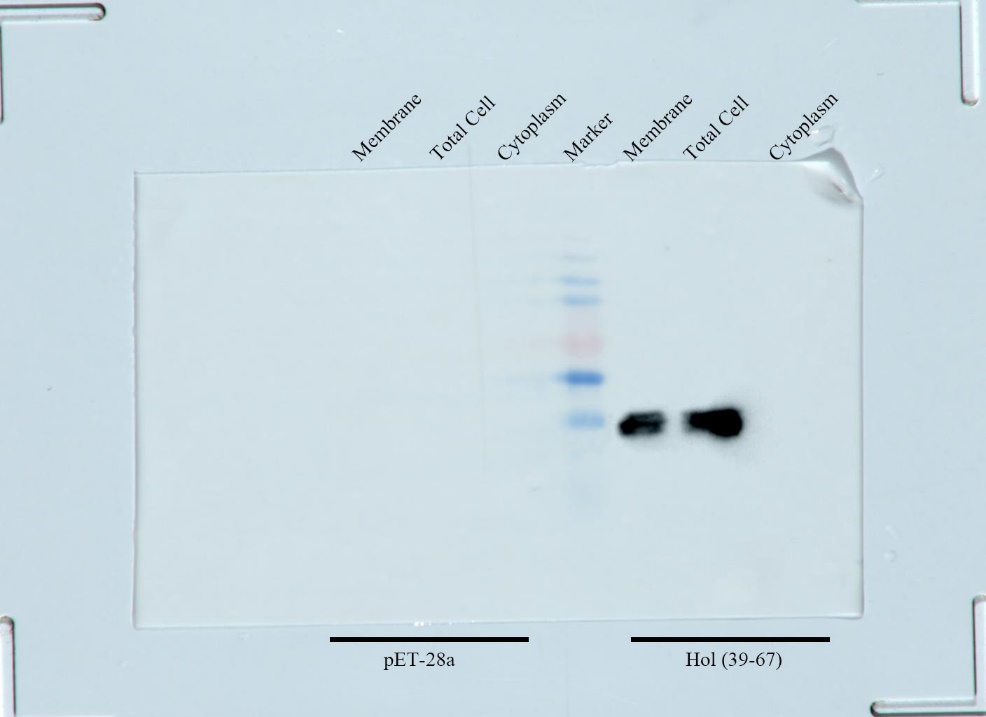


1. **WB image of Hol (61D), Hol (58D), Hol (63D), Hol (64D), Hol (65R), Hol (66R), Hol (61,63,64D), Hol (65,66R), Hol (63,64D).**


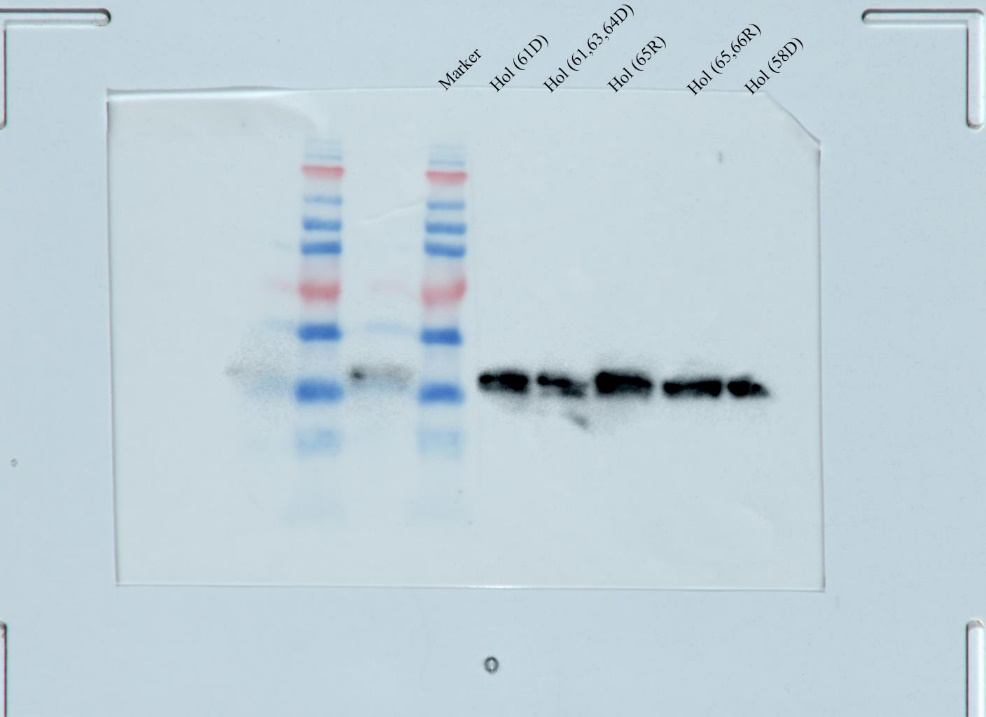

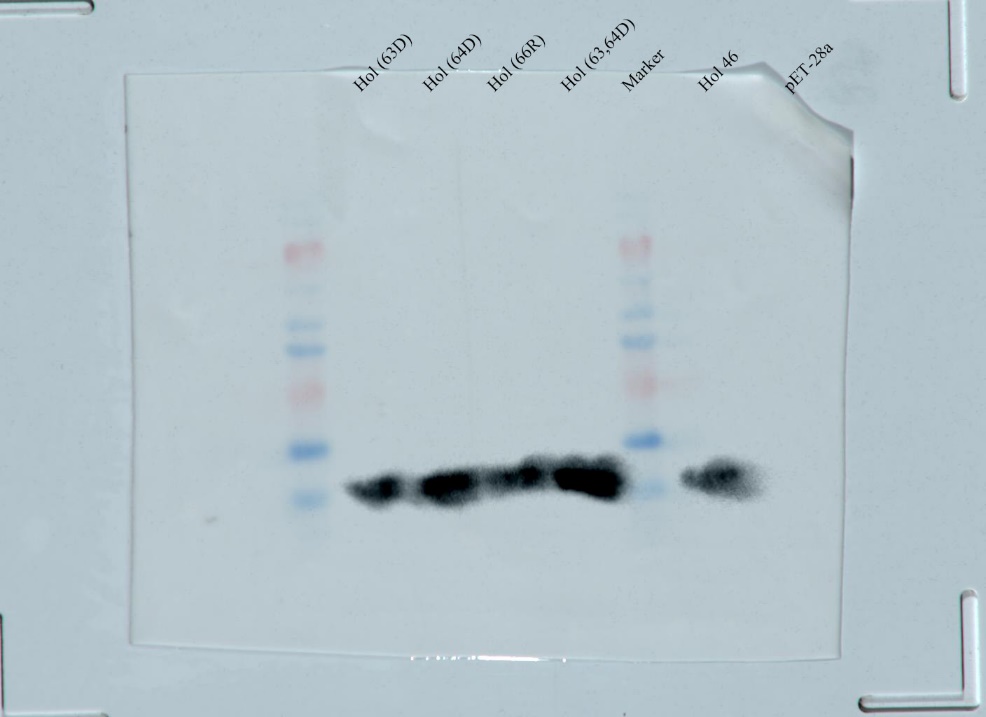


1. **WB image of Lys 17 protein**


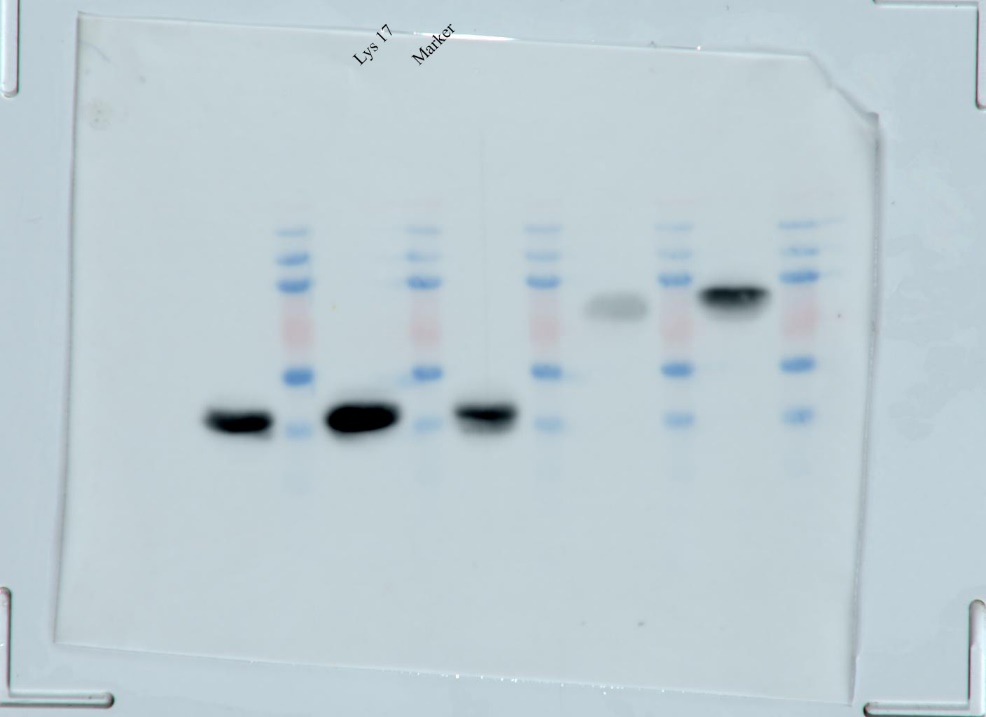


1. **WB image of Hol 46_Lys 17 protein**


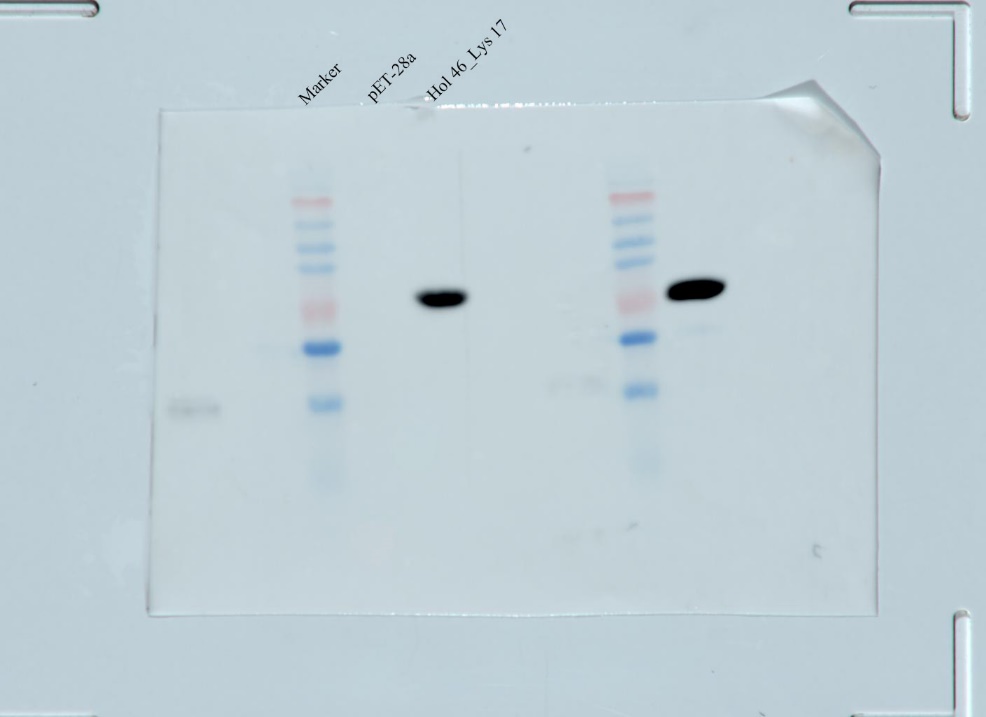


1. **WB image of Hol 46 protein with N-terminal and C-terminal double His tags (Hol 46 NC)**


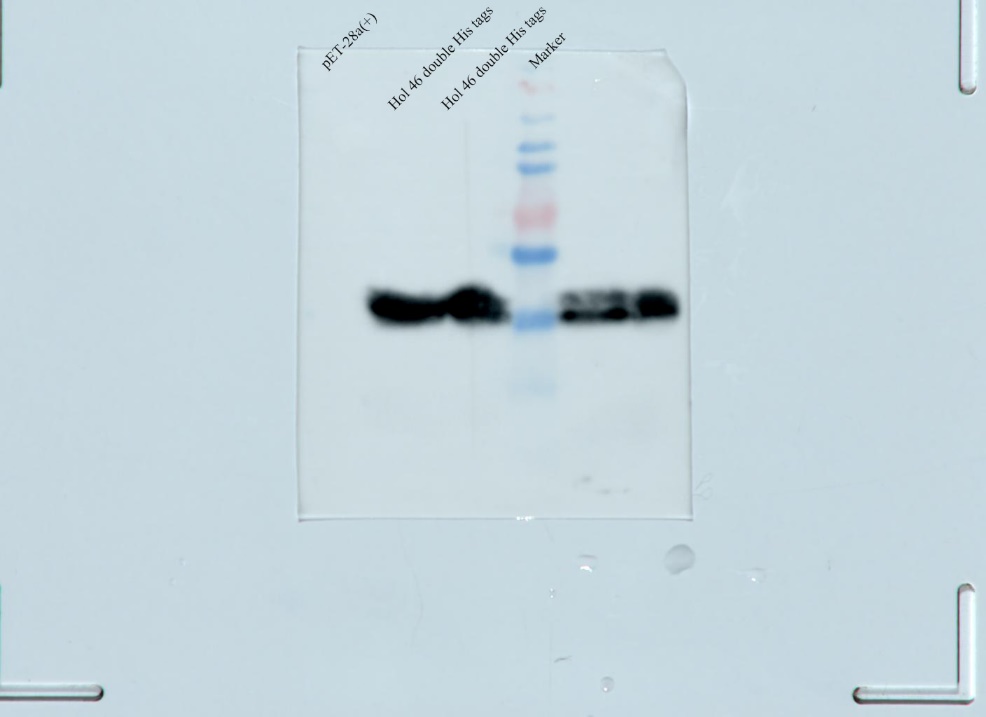


**I. WB image of S105 protein**


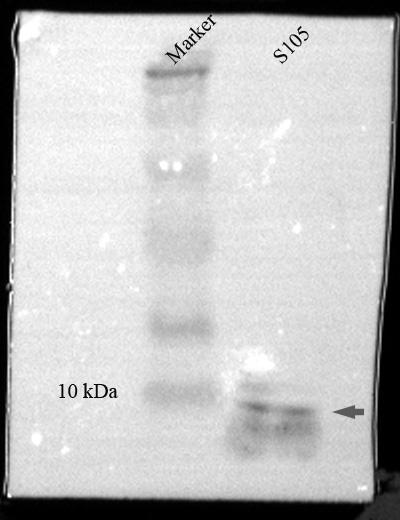

Supplement: Figure S1 — Uncut WB image. [file jvi.00832-25-s0001.docx]
