# Supplementary material for: Identification and analysis of the crucial holin domain and sites and the bactericidal activity of a holin–endolysin lysis cassette from phage PZL-Ah152 against Aeromonas hydrophila
Source: J Virol. 2025 Dec 15;100(1):e00832-25. doi: 10.1128/jvi.00832-25 (PMC12817945; doi:10.1128/jvi.00832-25)
Supplement: Table S1 — Primers for protein construction. [file jvi.00832-25-s0002.docx]

**Supplementary Table 1. Primers for protein construction**

| Gene name | Primers Sequence (5’- 3’) |
| --- | --- |
| *ORF 46* | F: tcgagctccgtcgacAAGCTTATGGCCCTTGAGACTTCACAGG  R: gtggtggtggtggtgCTCGAGCTATGTTTCCTCCTTCTTGAGTTTGA |
| *Hol 46 NC* | F: cagcaaatgggtcgcGGATCCATGGCCCTTGAGACTTCACAGG  R: gtggtggtggtggtgCTCGAGCTACACCACCACCACCACCAC |
| *GFP- Hol 46* | F: cagcaaatgggtcgcGGATCCATGGTGAGCAAGGGCGAGG  R: ttgtcgacggagctcGAATTCCTTGTACAGCTCGTCCATGCC |
| *Hol (1-57)* | F: atgggtcgcggatccGAATTCATGGCCCTTGAGACTTCACAGG  R: gtggtggtggtggtgCTCGAGCTATTTATACAATACGGCCCACACT |
| *Hol (39-67)* | F: atgggtcgcggatccGAATTCATGGAATGGTTTTACGTAGCGT  R: gtggtggtggtggtgCTCGAGCTATGTTTCCTCCTTCTTGAGTTT |
| *Hol (△TMD)* | F: atgggtcgcggatccGAATTCATGGCCCTTGAGACTTCACA  R: CTATGTTTCCTCCTTCTTGAGTTTGAGTGTTTTTTCGTTAAGTGT  2R: gtggtggtggtggtgCTCGAGCTATGTTTCCTCCTTCTTGAGT |
| *GFP-Hol (39-67)* | F: tcgagctccgtcgacAAGCTTATGGAATGGTTTTACGTAG  R: gtggtggtggtggtgCTCGAGCTATGTTTCCTCCTTCTTGA |
| *Hol (61D)* | F: AACACTCgacCTCAAGAAGGAGGAAACATAGCTCG  R: TCTTGAGgtcGAGTGTTTTATACAATACGGCCCA |
| *Hol (61,63,64D)* | F: ACTCgacctcgacgacGAGGAAACATAGCTCGAGCACC  R: TCgtcgtcgaggtcGAGTGTTTTATACAATACGGCCCA |
| *Hol (65R)* | F: CTCAAGAAGaggGAAACATAGCTCGAGCACCACC  R: GTTTCcctCTTCTTGAGTTTGAGTGTTTTATACAATACG |
| *Hol (65,66R)* | F: AGAAGaggaggACATAGCTCGAGCACCACCACC  R: GCTATGTcctcctCTTCTTGAGTTTGAGTGTTTTATACAATACG |
| *Hol (58D)* | F: GCCGTATTGTATgacACACTCAAACTCAAGAAGGAGGAAA  R: GTgtcATACAATACGGCCCACACTTGGACGAG |
| *Hol (63D)* | F: CAAACTCgacAAGGAGGAAACATAGCTCGAGCA  R: CCTCCTTgtcGAGTTTGAGTGTTTTATACAATACGGC |
| *Hol (64D)* | F: ACTCAAGgacGAGGAAACATAGCTCGAGCACC  R: TTTCCTCgtcCTTGAGTTTGAGTGTTTTATACAATACGG |
| *Hol (66R)* | F: GAAGGAGaggACATAGCTCGAGCACCACCACC  R: GCTATGTcctCTCCTTCTTGAGTTTGAGTGTTTTATACA |
| *Hol (63,64D)* | F: AAACTCgacgacGAGGAAACATAGCTCGAGCACC  R: TTCCTCgtcgtcGAGTTTGAGTGTTTTATACAATACGGC |
| *Hol 46_Lys 17* | F1: CAGCAAATGGGTCGCGGATCCATGGCCCTTGAGACTTCACA  R1: GCCGCTGCCGCTGCCGCTGCCTGTTTCCTCCTTCTTGAGTTTG  F2: GGCAGCGGCAGCGGCAGCGGCATGAAGGTGAAGTTCAAGAAG  R2: TCAGTGGTGGTGGTGGTGGTGCTCGAGTCAGCCAATGTCTTCCTC |
| *ORF 17* | F: cagcaaatgggtcgcGGATCCATGAAGGTGAAGTTCAAGAAGCG  R: gtggtggtggtggtgCTCGAGTCAGCCAATGTCTTCCTCCTTC |

Note：Lowercase letters denote homologous arm sequences (with lowercase nucleotides in mutagenesis primers indicating mutated bases).

**PCR reaction conditions**

| Sample | The usage（μL） |
| --- | --- |
| ddH_2_O | 7.5 |
| Forward primer | 1.0 |
| Reverse primer | 1.0 |
| 2 × Phanta Max Master Mix (Dye Plus) | 12.5 |
| DNA | 3.0 |
| Total | 25.0 |

PCR amplification was performed under the following conditions: initial denaturation at 94°C for 5 min; 30 cycles of denaturation at 94°C for 1 min, annealing at 71°C for 1 min, and extension at 70°C for 1 min; followed by a final extension at 72°C for 10 min, with samples held at 4°C. PCR products were electrophoresed on a 1% agarose gel and visualized to confirm expected band sizes.
