# Supplementary material for: Identification and analysis of the crucial holin domain and sites and the bactericidal activity of a holin–endolysin lysis cassette from phage PZL-Ah152 against Aeromonas hydrophila
Source: J Virol. 2025 Dec 15;100(1):e00832-25. doi: 10.1128/jvi.00832-25 (PMC12817945; doi:10.1128/jvi.00832-25)
Supplement: Table S2 — Sequences of primers used for real-time quantitative PCR. [file jvi.00832-25-s0003.docx]

**Supplementary Table 2. Sequences of Primers Used for Real-Time Quantitative PCR**

| Primers | Sequence (5’-3’) | Accession no. |
| --- | --- | --- |
| β-actin-F | TGGACTTTGAGCAGGAGATGG | AF079831.1 |
| β-actin-R | CTAGGAAGGATGGCTGGAAAA |  |
| TGF-β-F | TGATGTGCTTCAGTCTTTCCA | EU086521 |
| TGF-β-R | CATACCAATGCGACATACAAG |  |
| IL-10-F | GGAACGATGGGCAGATCAA | AY887900.1 |
| IL-10-R | AACTGAAGGGGAAGGGGAAG |  |
| ZO-1-F | GTTTGCCGTTGTAAAGGGTGT | HQ656016.1 |
| ZO-1-R | TGGCTCAGAGGAAGAAAGATG |  |
| Occludin-F | GGACGACTGGGCCATTGGGTA | HQ110086.1 |
| Occludin-R | CAGGCAGGGCAAGGGTTTCAT |  |
| Claudin4-F | GCTTTCTTATTCGCTGGCGG | XP_026134569.1 |
| Claudin4-R | TGATAAGCCATGGGTGCGTT |  |
| Muc2-F | TCAGCAGGGTGTCCATTTCC | XP_026057609.1 |
| Muc2-R | CACAAGGATCCCTGCGACAT |  |
| IL-1β-F  IL-1β-R | AACTGATGACCCGAATGGAAAC  CACCTTCTCCCAGTCGTCAAA | AY340959.1 |
| TNF-α-F  TNF-α-R | TTATGTCGGTGCGGCCTTC  AGGTCTTTCCGTTGTCGCTTT | AY427649.1 |
| IFN-γ-F  IFN-γ-R | AACAGTCGGGTGTCGCAAG  TCAGCAAACATACTCCCCA | EU909368.1 |
