# Supplementary material for: Identification and analysis of the crucial holin domain and sites and the bactericidal activity of a holin–endolysin lysis cassette from phage PZL-Ah152 against Aeromonas hydrophila
Source: J Virol. 2025 Dec 15;100(1):e00832-25. doi: 10.1128/jvi.00832-25 (PMC12817945; doi:10.1128/jvi.00832-25)
Supplement: Table S3 — Evaluation of the bactericidal activity of fusion protein Hol 46_Lys 17. [file jvi.00832-25-s0004.docx]

**Supplementary Table 3. Evaluation of the bactericidal activity of fusion protein Hol 46_Lys 17**

| **Bacterial strain** | **Name** | **Hol 46** | **Lys 17** | **Hol 46+ Lys 17** | **Hol 46_Lys 17** | **PZL-Ah152** |
| --- | --- | --- | --- | --- | --- | --- |
| *A. hydrophila* | Ah-138 | + | - | + | + | - |
|  | Ah-TPS | + | - | - | + | + |
|  | Ah-152 | + | - | + | + | + |
|  | Ah-BSK | - | - | - | + | - |
|  | 7966 | - | - | - | + | - |
|  | Ah-87 | + | - | - | + | + |
|  | Ah-JY22 | - | - | - | - | - |
|  | Ah-B3 | - | - | - | + | - |
|  | Ah-S60-2 | - | - | - | - | - |
|  | Ah-3-1 | - | - | - | - | - |
|  | Ah-NY7 | - | - | - | + | - |
|  | Ah-NY10 | - | - | - | - | - |
|  | Ah-23 | + | - | - | + | + |
|  | Ah-11 | - | - | - | - | - |
|  | Ah-57 | - | - | - | + | - |
|  | Ah-XLCJY2 | - | - | - | - | - |
|  | Ah-LHGY | - | - | - | + | - |
|  | Ah-JY1 | - | - | - | + | - |
|  | Ah-XLCJY5 | - | - | - | - | - |
|  | Ah-XLCJY1 | + | - | - | + | + |
|  | Ah-JY6 | - | - | - | - | - |
|  | Ah-JY7 | - | - | - | + | - |
|  | Ah-XLCJY4 | - | - | - | - | - |
|  | Ah-S72-1 | - | - | - | - | - |
|  | Ah-S71-6 | - | - | - | + | - |
|  | Ah-S71-4 | - | - | - | + | - |
|  | Ah-S57-7 | - | - | - | - | - |
|  | Ah-S28-6 | - | - | - | + | - |
| *A. veronii* | Th-0426 | - | - | - | + | - |
|  | Av-32 | - | - | - | + | - |
|  | Av-2 | - | - | - | + | - |
|  | Av-3 | - | - | - | + | - |
|  | Av-46 | - | - | - | + | - |
| *A. caviae* | ACCY | + | - | - | + | - |
|  | 15468 | + | - | - | + | - |
|  | AC-6 | - | - | - | - | - |
|  | AC-JY8 | - | - | - | - | - |
|  | AC-S23 | - | - | - | - | - |
| *Escherichia coli* | BL21 | + | - | - | + | - |
|  | DH-5α | + | - | + | + | - |
|  | 3-1 | - | - | - | - | - |
|  | 6-1 | - | - | - | - | - |
|  | 12-1 | - | - | - | - | - |
|  | 26-1 | - | - | - | - | - |
|  | 29-1 | - | - | - | - | - |
|  | 26-4-1 | - | - | - | - | - |
|  | F6-10-2 | - | - | - | - | - |
|  | 2-1 | - | - | - | - | - |
|  | 2-3 | - | - | - | - | - |
|  | F1-10-22 | - | - | - | + | - |
| *Salmonella* | 29 | - | - | - | + | - |
|  | SE-E-1 | - | - | - | + | - |
|  | M | - | - | - | - | - |
|  | CVCC2229 | - | - | - | - | - |
|  | CMCC50041 | - | - | - | + | - |
|  | G | - | - | - | + | - |
|  | 28 | - | - | - | - | - |
|  | 3775-F | - | - | - | - | - |
|  | N | - | - | - | - | - |
|  | 414 | - | - | - | - | - |
|  | B9 | - | - | - | - | - |
|  | L2 | - | - | - | - | - |
|  | JSST | - | - | - | - | - |
|  | L5 | - | - | - | - | - |
|  | CMCL | - | - | - | - | - |
|  | P | - | - | - | - | - |
|  | 14028 | - | - | - | + | - |
|  | YLA | - | - | - | - | - |
|  | JSSTV | - | - | - | - | - |
|  | S1 | - | - | - | - | - |
|  | S2 | - | - | - | - | - |
|  | S3 | - | - | - | - | - |
|  | S4 | - | - | - | - | - |
|  | S5 | - | - | - | - | - |
|  | S6 | - | - | - | - | - |
|  | S7 | - | - | - | - | - |
|  | S8 | - | - | - | - | - |
|  | S9 | - | - | - | - | - |
|  | S10 | - | - | - | - | - |

Note: '+' indicates sensitive, and '-' indicates non-sensitive.
