# Supplementary material for: Identification and analysis of the crucial holin domain and sites and the bactericidal activity of a holin–endolysin lysis cassette from phage PZL-Ah152 against Aeromonas hydrophila
Source: J Virol. 2025 Dec 15;100(1):e00832-25. doi: 10.1128/jvi.00832-25 (PMC12817945; doi:10.1128/jvi.00832-25)
Supplement: Table S4 — RT-qPCR primers for transcriptome sequencing. [file jvi.00832-25-s0005.docx]

**Supplementary Table 4. RT-qPCR primers for transcriptome sequencing**

| Gene ID | Gene name | Primers Sequence (5’- 3’) |
| --- | --- | --- |
| AHA_RS06870 | *fliF* | F: GCGGTGGATTACAAGGCGTCTAC  R: ATCACCACGGGTCACATCAAAGC |
| AHA_RS06885 | *fliI* | F: TGGTGGTGGTGGGGCTCATC  R: GCATCAGCGGCGAGGCATC |
| AHA_RS06895 | *fliK* | F: GCTGCCGCTGCCGACAAG  R: CTGCTGCACCCGGTTCATCAC |
| AHA_RS06940 | *flhA* | F: CAACATCCTGGGCGGCTTCATC  R: TGGCGGCGGCGATGGAG |
| AHA_RS14340 | *flgG* | F: GCGTGACGATTGGCGAGGATG  R: CGAAGTCGGTGGTGTTGATCTGG |
| AHA_RS14335 | *flgH* | F: AACGGCATCTATTCGGACATCAAGG  R: TCTTGGAGGCGGAAGTGGACTC |
| AHA_RS14275 | *flrC* | F: GTGCTGTTGATGACCGCCTACG  R: CACATAACGGCTGACCTGGTTGAG |
| AHA_RS06955 | *fliA* | F: GATGAAGAACTGAACTTGCGTGAAATAG  R: AATCTGTGCATCGCCTGACTATGTATC |
| AHA_RS05505 | *flgP* | F: ACTCGCTGGACGTGTCGGTAG  R: CCATCTCGGTGGCATAGGTGTTG |
| AHA_RS03320 | *motX* | F: GCAGCAGGTGAAGCAGGACAG  R: CTCCGCATTGGGCTTGATACAGAC |
| AHA_RS13345 | *motY* | F: GCCTTCAGCGTGCTCACCTATG  R: CGTCGATGCTCTTGTCCACCTTC |
| AHA_RS14285 | *flrA* | F: TACGAATCCCTGACCCGCCATC  R: TGAGCCGACGCACCTCCTG |
| AHA_RS06875 | *fliG* | F: TGCTGGCGGACGATCTGGAG  R: CGGCGGGCGATGGAGAGG |
| AHA_RS06905 | *fliM* | F: GTGCCGACCAGCCTGAACATG  R: CGCCACCGAAGAAGTTCTCCAC |
| AHA_RS06910 | *fliN* | F: GCCAGATCAGCATCCGCAACC  R: CTTGTCGTTCACCACCACTACCTC |
| AHA_RS06985 | *motB* | F: CTCTATTCGGTGGCACTGGTCAAC  R: GGCGGCAGATACCTCGTTGTTC |
| AHA_RS16310 | *qseC* | F: TCGTGGCGGAGCGGGATG  R: AGCAGGAAGGGCAGGGTGATG |
| AHA_RS00070 | *kdpD* | F: GCAGTCCTTCTTCTCCCTCAACAAC  R: GCAGCGGCAGTGTGGTCTTG |
| AHA_RS00065 | *kdpE* | F: GCTATCGGCACGGGATTCTGAAC  R: CCGAACGGCTTGCTCATGTAGTC |
| AHA_RS00085 | *kdpA* | F: CGTGCTGCTGACCGTGTTCC  R: AATGCCGCCGATCACCAATACC |
| AHA_RS00080 | *kdpB* | F: CCAGCCCGCAAGATGACAAGG  R: GGTGATGGCAGCCTCGTTGAC |
| AHA_RS00075 | *kdpC* | F: TGCTGACCCTGACCCTGCTG  R: TGCTGCTTGGCGTTGGTGAG |
